# Viscous shear is a key force in *Drosophila* ventral furrow morphogenesis

**DOI:** 10.1242/dev.202892

**Published:** 2024-11-15

**Authors:** Amanda Nicole Goldner, Mohamad Ibrahim Cheikh, Miriam Osterfield, Konstantin Doubrovinski

**Affiliations:** ^1^ Department of Biophysics, University of Texas Southwestern Medical Center, 5323 Harry Hines Boulevard, Dallas, TX 75390, USA; ^2^ Department of Cell Biology, University of Texas Southwestern Medical Center, 5323 Harry Hines Boulevard, Dallas, TX 75390, USA

**Keywords:** Gastrulation, Morphogenesis, Anillin, *Drosophila*, Shear, Viscosity

## Abstract

Ventral furrow (VF) formation in *Drosophila melanogaster* is an important model of epithelial folding. Previous models of VF formation require cell volume conservation to convert apically localized constriction forces into lateral cell elongation and tissue folding. Here, we have investigated embryonic morphogenesis in anillin knockdown (*scra* RNAi) embryos, where basal cell membranes fail to form and therefore cells can lose cytoplasmic volume through their basal side. Surprisingly, the mesoderm elongation and subsequent folding that comprise VF formation occurred essentially normally. We hypothesized that the effects of viscous shear may be sufficient to drive membrane elongation, providing effective volume conservation, and thus driving tissue folding. Since this hypothesis may not be possible to test experimentally, we turned to a computational approach. To test whether viscous shear is a dominant force for morphogenesis *in vivo*, we developed a 3D computational model incorporating both accurate cell and tissue geometry, and experimentally measured material parameters. Results from this model demonstrate that viscous shear generates sufficient force to drive cell elongation and tissue folding *in vivo*.

## Teaser

**Summary:** Examination of *Drosophila* mutants in which basal membranes fail to form reveals a role for viscous forces in epithelial folding.

## INTRODUCTION

Epithelial folding occurs throughout many stages in animal development (Leptin, 1991; Martinez Arias and Stewart, 2003), including vertebrate neurulation (Maruyama and Andrew, 2012), lung bud formation (Kim et al., 2013) and *Drosophila* ventral furrow (VF) formation (Campos-Ortega and Hartenstein, 1985; Kohler, 1994). The *Drosophila* VF is an ideal model system to address the physical mechanisms underlying epithelial folding, since it has been highly characterized genetically and morphologically (Barrett et al., 1997; Costa et al., 1994; Kanesaki et al., 2013; Kerridge et al., 2016; Kölsch et al., 2007; Lye and Sanson, 2011; Manning et al., 2013; Sweeton et al., 1991; Turner and Mahowald, 1977).

Several physical mechanisms of VF formation have been proposed over the past few years, and while these models differ in important details, they all assume two common requirements: (1) apical constriction and (2) volume conservation of cells (Fierling et al., 2022; Heer et al., 2017; Perez-Mockus et al., 2017; Polyakov et al., 2014; Rauzi et al., 2013). The first of these points has been strongly supported experimentally (Dawes-Hoang et al., 2005; Izquierdo et al., 2018; Sawyer et al., 2010), but the second of these assumptions has only recently begun to be questioned or tested. A recent paper from Matteo Rauzi's lab (Fierling et al., 2022) demonstrated that an acellular embryo (which has no cell volume to conserve) can form a small surface-level furrow, but tissue invagination fails. They attribute the initial, superficial furrow to buckling forces, and suggest that invagination may require cytoplasmic compartmentalization (although additional factors associated with the genetic background of the acellular embryo may also be contributing to the phenotype). In this work, we directly test whether cellular compartmentalization is indeed required for invagination.

First, we begin by reviewing the developmental processes that precede and set the stage for gastrulation in the fruit fly. After fertilization of a *Drosophila* egg, the nuclei undergo 13 division cycles and migrate to the periphery of the embryo. Since nuclear division at this stage is not accompanied by cytokinesis, this results in an embryo that is essentially a large multinucleate cell encased in a rigid vitelline membrane (Leptin, 1991; Zalokar and Erk, 1976). Subsequently, lateral membranes grow inwards to compartmentalize peripheral nuclei into individual cells, forming the epithelium in a process known as cellularization (Campos-Ortega and Hartenstein, 1985; Lecuit and Wieschaus, 2000). Throughout the course of cellularization, cells remain open to the yolk sac such that there is a ‘hole’ on the basal side of each cell (see schematic in Fig. S1) (Krueger et al., 2019; Loncar and Singer, 1995; Mazumdar and Mazumdar, 2002).

As cellularization completes, gastrulation begins with the folding of the ventral furrow (VF) (Campos-Ortega and Hartenstein, 1985; Leptin and Grunewald, 1990; Sweeton et al., 1991; Turner and Mahowald, 1977). VF formation involves a sequence of shape changes in a subset of ventral cells, starting with constriction of their apical membranes (Kam et al., 1991; Leptin, 1991). As VF formation proceeds, lateral membranes lengthen before eventually shortening again as the basal membranes seal and the ventral tissue invaginates to form a furrow (Lye and Sanson, 2011; Sweeton et al., 1991; Turner and Mahowald, 1977). Initially, these shape changes were thought to be driven primarily by apical acto-myosin constriction (Martin et al., 2009, 2010; Sawyer et al., 2010), although more recent work has indicated that lateral tensions may also play an important role (Gracia et al., 2019).

In this study, we show that basal membranes are not required for epithelial folding in the VF. This is a very surprising finding, given the following observations: (1) all previously proposed models of VF formation absolutely require cell volume conservation for tissue invagination to occur; and (2) cytoplasm in the early *Drosophila* embryo is viscous, not visco-elastic or elastic (Doubrovinski et al., 2017; He et al., 2014; Selvaggi et al., 2018), suggesting that basally open cells should in fact lose volume when compressed by myosin-generated forces. Hence, understanding the mechanism of ventral furrow formation requires explaining what force confines the cytoplasm (and other cellular structures) to cellular interiors when basal membranes no longer separate those cellular interiors from the adjacent yolk sack. We therefore computationally explored alternative mechanisms for folding and found that viscous shear can compensate for the lack of basal membranes to maintain cell volume conservation and to drive tissue folding. That is, if sufficiently viscous, the cytoplasm cannot empty out into the yolk sack through open basal holes during the relatively short course of VF formation, and cell volume is thus maintained, even though basal membranes are missing. We then used a 3D model with experimentally measured parameters to test this model quantitatively, and found that this mechanism indeed operates in the time-scales and material property parameter regimes found *in vivo*.

## RESULTS

### Basal membranes are not required for VF formation

**Fig. 1.**
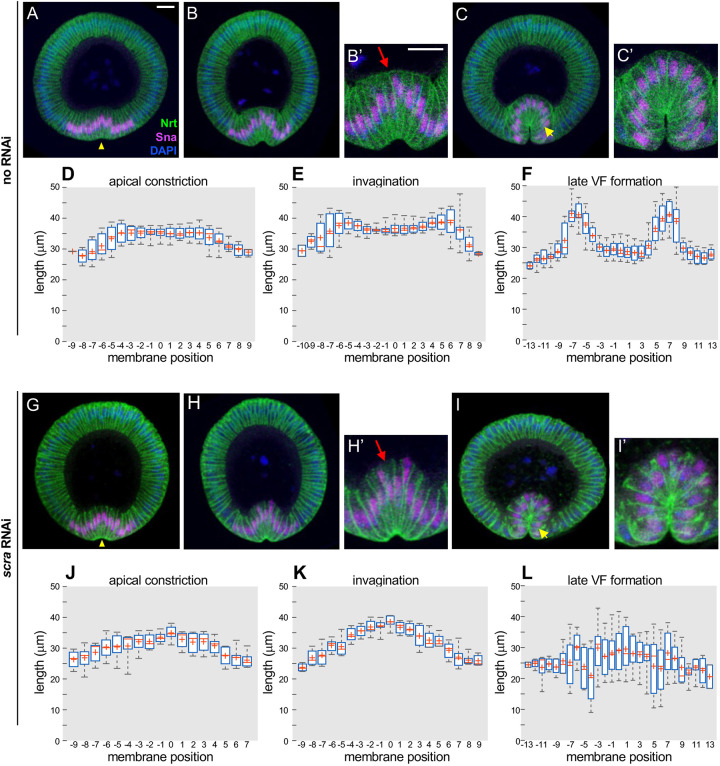
**Basal membranes are not required for VF formation.** Confocal immunofluorescence (A-C′,G-I′) and quantification of tissue morphology (D-F,J-L) in controls lacking scraTRiP (A-F) and in *scra* RNAi (G-L) embryos. (D-F,J-L) The *x*-axis in the plot is the membrane position counted as the number of cells between it and the ventral midline, with negative numbers to the left and positive to the right; the ventral midline is identified as the center of the pattern of Snail expression. (A,G) Embryos at apical constriction stage. Yellow arrowheads indicate the apical surface of ventral mesoderm cells, which are constricting in both genetic backgrounds. (B,H) Embryos at early invagination stage. Ventrally located (Snail positive) mesoderm cells elongate along their apical-basal axis in both genotypes, and tissue invagination has begun. Basal surfaces, indicated by red arrows in B′ and H′, are closed in control but remain open in *scra* RNAi embryos. (C,I) Embryos at late VF stage. Mesodermal cells have fully invaginated into the interior of the embryo in both genotypes, although basal surfaces still remain open in *scra* RNAi embryos. Yellow arrows indicate the peripheral VF cells, which are shorter in *scra* RNAi than in control. All images shown were cropped, rotated ventral side down and set against a black background. (C′,I′) Higher magnifications of C,I. Scale bars: 20 µm. (D-F) Mesoderm lateral membrane lengths in control embryos during (D) apical constriction (*n*=9 embryos), (E) invagination (*n*=7 embryos) and (F) late VF formation (*n*=10 embryos). (J-L) Mesoderm lateral membrane lengths in *scra* RNAi embryos during (J) apical constriction (*n*=8 embryos), (K) invagination (*n*=8 embryos) and (L) late VF formation (*n*=6 embryos). (B′,C′,H′,I′) 2× magnifications of B,C,H,I, respectively. Data are represented as box and whisker plots, where the central mark is the median, the cross is the mean, and the box edges are the 25th and 75th percentiles. Although control and *scra* RNAi embryos appear qualitatively similar at the level of gross tissue morphology, lateral membrane lengths at each of the three stages of VF formation are significantly different between these two genotypes by the two-sample Kolmogorov Smirnov test (wild type versus *scra* RNAi, *P*<0.001); see the section ‘Statistical analysis of immunostaining results’ for further details. See also Figs S2-S6.

We began by exploring the role of basal membranes in VF formation. Loss-of-function mutations in Anillin (Scraps) – a component of the contractile ring scaffold that narrows to seal off cells and form basal membranes – delay basal membrane formation (Field et al., 2005; Thomas and Wieschaus, 2004; Xue and Sokac, 2016). Anillin protein localizes specifically to the leading (i.e. basal) edges of the lateral membranes as they grow longer then inwards to compartmentalize the cells (Field et al., 2005; Thomas and Wieschaus, 2004), and loss of anillin results in loss of actin, myosin and septin from these structures (Field et al., 2005). However, gastrulation has not been characterized in this genetic background. For experimental simplicity, we first replicated this phenotype via maternal GAL4-driven expression of short hairpin RNA against *scra* (UAS-scraTRiP). Live imaging of embryos expressing maternal GAL4, UAS-scraTRiP and a membrane marker (UAS-Nrt-GFP) showed that the effects of *scra* RNAi during cellularization were indistinguishable from those seen in Anillin loss-of-function mutants (Fig. S2A,B) (Field et al., 2005; Thomas and Wieschaus, 2004; Xue and Sokac, 2016). To better characterize gastrulation in this background, we prepared sections of heat-fixed gastrulas and stained them for Neurotactin (Nrt; a membrane marker), Snail (Sna; a mesoderm marker) and DAPI to assess morphology (Fig. 1). As a control, embryos of similar genetic background without UAS-scraTRiP were stained according to the same protocol (Fig. 1A-C); additional experiments with a non-relevant TRiP/RNAi line substituted for the UAS-scraTRiP showed similar results to the no RNAi control (Fig. S2C,D). Although basal membranes never form in *scra* RNAi gastrulas, the VF consistently folds to its usual depth (Fig. 1G-I).

### Membrane lengths are altered in *scra* RNAi embryos

We were interested to see whether there were any morphological differences between *scra* RNAi and control embryos. To this end, we compared the distribution of lateral membrane lengths along transverse sections of control (Fig. 1D-F) and *scra* RNAi (Fig. 1J-L) embryos at various stages of VF formation, first confining our comparisons to mesoderm (Snail-positive) cells. In both genetic backgrounds, lateral membranes lengthen as apical constriction proceeds into invagination, then shorten as the VF forms more fully (Fig. 1D-F,J-L). However, the spatial pattern of membrane lengths diverges in these genetic backgrounds as the VF forms. During early invagination, the peripheral mesoderm cells lengthen, while the equivalent *scra* RNAi cells remain essentially unchanged in length (Fig. S3). Later, as the VF fully forms, the longest lateral membranes are located in the center of the furrow in *scra* RNAi embryos (single peak in Fig. 1L), while the longest membranes in the control embryos are located peripherally within the VF (two peaks in Fig. 1F). In particular, the membranes in the peripheral regions of the VF are strikingly shorter in *scra* RNAi embryos than in controls (yellow arrows in Fig. 1C,I, quantified in Fig. 1F,L and Fig. S4A). This observation is consistent with a loss of basal tension in *scra* RNAi embryos, since higher basal tension (found in control) would tend to reduce the overall curvature of the basal surface. This effect will be strongest where basal membrane curvature is highest, i.e. at the location of the peripheral VF cells. In the control, basal tension will stretch those peripheral VF cells along the apico-basal direction. In the *scra* RNAi background, this effect is expected to be weaker since basal tension is likely reduced, allowing the peripheral VF cells to shorten to a greater extent compared to the control.

We also measured the length of ectodermal membranes (outside the VF) at the same three stages of VF formation (Fig. S5). In both cases, ectodermal membranes lengthen during VF formation. However, ectodermal lengthening is delayed in *scra* RNAi embryos (Fig. S5).

### *scra* RNAi VFs remain folded despite degradation of lateral membranes

Anillin mutants additionally exhibit membrane degradation and nuclear displacement (Field et al., 2005). Depletion of anillin in *scra* RNAi gastrulas also results in the gradual disintegration of lateral membranes, which eventually form vesicles (Fig. S6). Membrane degradation increases in severity over time. Some VF cells become multinucleated, and VF nuclei are intermittently displaced into the yolk sac (Fig. 1H′,I′, Fig. S6). These findings suggest that Anillin affects cell behavior during VF formation – not only during actin ring closure and basal membrane formation, but also in maintaining membrane stability.

**Fig. 2.**
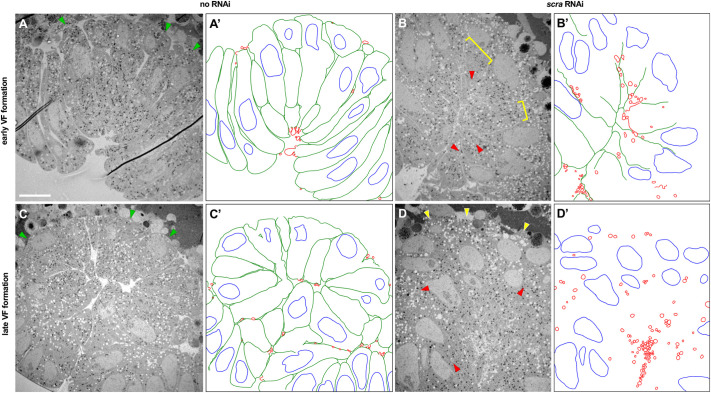
***scra* RNAi VFs remain folded, despite degradation of lateral membranes.** (A-D) Low magnification transmission electron micrographs of embryo sections prepared using a combined high pressure freezing/freeze substitution method during invagination (A,B) or late VF formation (C,D) in control (A,C) and *scra* RNAi (B,D) embryos. Interstitial spaces are visible on the basal sides of VF cells (green arrowheads) in control embryos during both early (A) and late (C) VF formation. Basal interstitial spaces are absent (yellow brackets) and lateral membranes are starting to degrade into vesicles (red arrowheads) in early VF formation *scra* RNAi embryos (B). Basal interstitial spaces are still absent (yellow arrowheads) during late VF formation in *scra* RNAi embryos (D) and lateral interstitial spaces are no longer visible, replaced by an increased number of vesicles (red arrowheads). (A′-D′) Hand-drawn traces of A-D, respectively, highlighting intact interstitial spaces (green), nuclei (blue) and vesicles (red). Scale bar: 10 μm.

The possibility remained that the phenotypes seen in immunostainings reflected differences in the localization of our Neurotactin membrane marker, rather than degradation or loss of actual membranes. To substantiate our findings, we examined the fine structure of epithelial membranes using transmission electron microscopy (TEM) (Fig. 2). At early stages of VF formation, interstitial spaces between ventral cells (indicating the presence of membranes) are visible in control embryos at low magnification (Fig. 2A) indicating the presence of intact membranes (traced in green). In early stage *scra* RNAi gastrulas, these interstitial spaces are less intact, and lateral membranes have started disintegrating into vesicles (traced in red) (Fig. 2B). Ventral cells in late stage control gastrulas have intact lateral and basal membranes (Fig. 2C), all of which are absent in late stage *scra* RNAi gastrulas (Fig. 2D). Specifically, at this late stage, interstitial spaces are no longer visible and have been replaced by an increased number of vesicles (Fig. 2D); in other words, lateral membranes have been lost. Together, these data substantiate our findings with Neurotactin immunostaining, and indicate that basal membranes never form in *scra* RNAi embryos, and lateral membranes degrade late in VF formation.

In spite of the gradual loss of membrane integrity during late stages of VF formation, the mesoderm nuclei maintain their ring-like spatial distribution. This indicates that the furrow retains its shape in the absence of basal, and subsequently lateral, membranes.

### Cellular cytoplasm is viscous

Many existing models of VF formation require volume conservation within cells (Rauzi et al., 2013). It is unclear how such models could describe a mutant in which basal membranes are missing. This is particularly puzzling if the embryonic cytoplasm is entirely viscous, and might therefore be capable of flowing out of apically constricting cells through the basal opening.

In several recent works, our lab and others have demonstrated that embryonic cytoplasm behaves as a viscous fluid and entirely lacks any measurable elasticity (Doubrovinski et al., 2017; He et al., 2014; Selvaggi et al., 2018). However, in those experiments, for experimental convenience, the cytoplasm was probed before its partitioning into individual cells. It is therefore possible that the material properties of the cytoplasm change as it is being partitioned into the interiors of the blastocyst. To test this possibility, we developed a new experimental protocol that allowed us to probe material properties of cellular interiors with subcellular spatial precision. First, using a fire-polished holding pipette, a magnetic microsphere is introduced into the yolk sack and placed near the basal hole that separates the blastocyst from the yolk. Next, an externally applied magnetic field is used to force the microsphere into the cell through the basal hole. As shown in Fig. S7 and Movie 1, the microsphere can be pulled all the way from the basal to the apical side of a cell. When the magnet is removed, the microsphere remains at the apical side, consistently showing no detectable recoil after the removal of the magnet (*n*=5, see Movie 1). This result strongly suggests that cellular interiors are viscous, since no elastic response (recoil of the microsphere) is seen. It is technically possible that the microsphere is too small to come into contact with elastic structures in the cellular interior such that it moves between those structures without causing them to deform. However, this is unlikely, since in our experiments, the radius of the bead was approximately half the diameter of a cell. Therefore, these experiments support the assumption that the cellular interior is viscous. However, one possible complication with the interpretation of the above bead-pulling experiment is the potential effect of yield stress: the bead might disrupt elastic structures in its path. In this way, the experiment may fail to reveal cytoplasmic elasticity. To test this possibility further, we performed additional measurements aimed at quantifying diffusion, where fluorescent polystyrene beads with a diameter of 0.5 µm were injected into embryos during cellularization stage. Samples were imaged for 10 min using confocal spinning disk microscopy. Individual particles within the cellular layer were then tracked and the tracks were analyzed by plotting their mean square displacement (MSD, 〈*r*^2^(*τ* )〉) as a function of time (Fig. S8). At 10 min, the MSD is approximately 18 µm^2^, implying that the average displacement of a bead after 10 min is √18=4.2 µm, i.e. ∼20% of cell height. More importantly, the MSD increases approximately linearly during the entire time-interval of 10 min, which is consistent with diffusion in a viscous medium. (In contrast, diffusion in an elastic medium results in a sub-linear MSD increase.) These data strongly indicate that the cytoplasm is viscous and does not exhibit appreciable elasticity, although it is formally possible that random forces constantly present within the cellular cytoplasm are sufficient to overcome yield stress.

### VF formation without basal membranes can be explained by viscosity

A mechanistic question remains: how are *scra* RNAi embryos capable of forming a furrow while cells remain open? To explore this question, we first developed a 2D model representing a transverse cross-section of the embryonic epithelium. In the model, the vitelline membrane is represented as a circular no-slip boundary encasing the model tissue. The perivitelline fluid, which fills the narrow space between the vitelline membrane and embryo, is modeled as a Newtonian fluid of fixed (low) viscosity. Cell membranes are modeled as series of short elastic springs. When we refer to ‘membranes’ in this section, we actually are referring to the membrane along with the associated load-bearing cytoskeleton. The yolk and cytoplasm within the embryo is modeled as a Newtonian fluid of viscosity η. In all of our modeling work, the volumes of the cells, of the yolk compartment and of the perivitelline space are individually conserved.

To model ventral furrow formation, we applied contractile stresses in a pattern chosen to mimic the forces thought to be present *in vivo* (Dawes-Hoang et al., 2005; Gracia et al., 2019; Leptin et al., 1992). Specifically, we assume that all membranes are subjected to a relatively small amount of constitutive stress, while the 16 mesodermal cells have additional stress applied to their apical and lateral membranes; this additional stress is linearly ramped up over time.

**Fig. 3.**
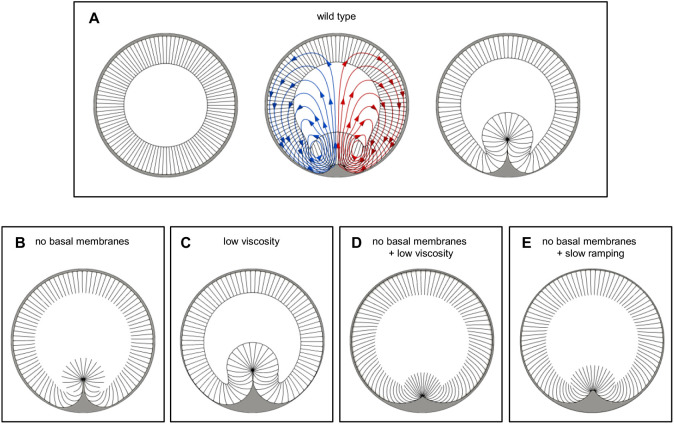
**Simplified two-dimensional model of VF formation.** (A) Two-dimensional model of VF formation. The initial state of the tissue is shown on the left. Middle panel shows a transient intermediate state of tissue during invagination; instantaneous fluid flow lines are also shown in red and blue. Right panel is the final state of the furrow (once maximal invagination depth has been reached, see Materials and Methods). (B-E) Final states of similar simulations in which specific material parameters or features have been altered. (B) Same parameters as in A, without the basal membranes. Cytoplasm can move between the cellular interiors and the yolk sack unobstructed. VF still forms successfully. (C) Same parameters as in A, except with the value of cytoplasmic/yolk viscosity reduced 100-fold. VF forms successfully. (D) Same parameters as in A, except without the basal membranes and with the value of the viscosity reduced 100-fold. The depth of VF invagination is markedly reduced. (E) Same parameters as in B, except that active stresses are ramped up 33-fold more slowly. The depth of VF invagination is markedly reduced. Changing cytoplasmic viscosity is (mathematically) equivalent to changing the time-scale of force ramping: changing either of those two quantities ultimately changes the ratio of the characteristic time of the mechanical tissue response and the time on which the active force attains its final value. We conclude that VF invagination requires cell volume conservation either due to the presence of the basal membranes (as in the control case) or due to the presence of sufficient viscous shear forces confining the cytoplasm to the cellular interior in the absence of the basal membranes (as in the *scra* RNAi background).

In the version of the model representing wild-type embryos, the mesoderm constricts apically and then invaginates as expected (Fig. 3A). When basal membranes are removed, mesoderm invagination still occurs (Fig. 3B), mimicking our observations in *scra* RNAi embryos. We had wondered how cytoplasmic contents remain within the interior of open cells in *scra* RNAi mutants; this model gave us an opportunity to explore the physical mechanisms underlying this phenomenon.

The reason we explicitly included fluid in our model is that we intuitively felt that shear (or viscous) forces may be responsible for invagination in *scra* RNAi mutants. Specifically, we hypothesized that shear forces imposed an effective volume constraint that prevented fluid from quickly leaving cells as they deformed. To test this hypothesis in our model, we reduced the value of η (the yolk and cytoplasmic viscosity) by 100-fold. With basal membranes present, apical constriction and mesoderm invagination occur normally (Fig. 3C), but in the absence of basal membranes, mesoderm invagination is greatly reduced (Fig. 3D). Furthermore, this reduction is accompanied by a shortening of lateral membranes and an apparent reduction of cytoplasmic volume within the mesoderm. These results support our hypothesis that shear forces play a key role in maintaining some degree of volume conservation in partially open cells during this morphogenetic movement.

Shear forces are a product of two factors: the viscosity of the fluid and the velocity gradient (which in the case of fluid moving between two surfaces is proportional to velocity itself). Having already tested the effect of lowering viscosity (Fig. 3D), we also examined the effects of changing the rate at which we ramped up contractile stresses in the mesoderm, since slower ramping would be expected to yield lower velocities and thus lower shear forces. We greatly decreased the ramping speed (by 33-fold) and allowed the simulation to run longer (to reach the same final values for stress); this again resulted in greatly reduced mesoderm invagination (Fig. 3E). This result confirmed that shear forces are required for invagination in models with basally open cells.

Importantly, these results also show that the parameters of the model affect the qualitative outcome: changing either the viscosity of the fluid or the ramping rate of the applied stresses can greatly affect the extent of invagination. This highlights the importance of considering a realistic, physically accurate model if we want use it to explore physical effects that are actually relevant to the biological system.

The 2D model considered above is useful for exploring general physical effects, but is more toy model than realistic representation. In particular, the parameters used were not based on measured values and were in fact in arbitrary units. (The exact 3D counterpart of the 2D model is a situation where cells are infinitely long along the antero-posterior axis of the embryo, which is, of course, not physiologically achievable cellular geometry.)

However, we have recently developed a 3D model of the *Drosophila* embryo that incorporates experimentally measured geometries and material properties (Cheikh et al., 2023). In particular, the cytoplasmic and yolk viscosity, and the Young's (elastic) moduli of the apical and basal-lateral membranes were all measured or estimated from experimental data (Cheikh et al., 2023; Doubrovinski et al., 2017) (see also Materials and Methods section). To address whether shear stresses are relevant to ventral furrow formation *in vivo*, we therefore asked to what extent shear stresses affect ventral furrow formation using this 3D model.

**Fig. 4.**
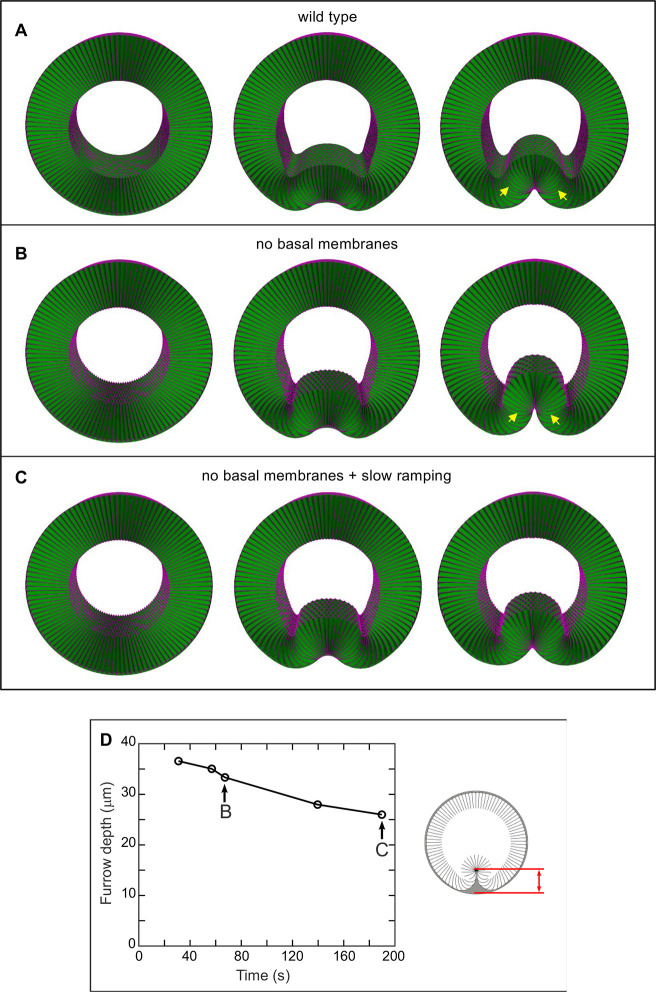
**Three-dimensional model of VF formation based on *in vivo* measurements.** (A) VF formation with basal membranes present (wild-type case). Left panel is starting configuration, middle panel is an intermediate time point and right panel is the final state of the furrow (once maximal invagination depth has been reached, see Materials and Methods). (B) Same as A, without the basal membranes. The VF forms successfully. Cells in the peripheral regions of the VF (arrows) are significantly shorter than the corresponding cells in A (quantified in Fig. S4). (C) Same as B, except that active stresses are ramped up threefold more slowly. Maximal invagination is reached approximately 3 min into force ramping (compared with ∼1 min in both A and B). (D) Simulations of tissue without basal membranes were run using a variety of ramping speeds. For each simulation, the maximal furrow depth (see red arrow in schematic on the right) is plotted as a function of the time taken to reach maximal VF invagination. Indicated points correspond to the simulations shown in B and C. Model parameters and simulation procedures are provided in the supplementary Materials and Methods.

To study VF formation in our 3D model, we again linearly ramped up apical and lateral membrane stress over time in the mesodermal cells. For a range of ramping speeds, mesoderm invagination appeared relatively normal in cases both with basal membranes (equivalent to wild type) and without basal membranes (equivalent to *scra* RNAi mutants) (Fig. 4). However, ramping speeds did affect the depth of furrow formation in the *scra* RNAi mutant case (Fig. 4D). For example, a ramping speed that reached maximal furrow depth at around 1 min (Fig. 4B) had a furrow depth of 33 µm, while a 300% slower ramping speed resulted in a final depth of 26 µm at around 3 min (Fig. 4C). The exact distribution of contractile forces is not known experimentally. However, we do not expect our key conclusions to be sensitive to the exact spatial distribution of those force, but only to the speed at which those forces are ramped.

*In vivo*, ventral furrow formation occurs over ∼5 to 10 min (Gelbart et al., 2012). This is slightly longer than the time scale on which we start to see decreasing maximal furrow depths in our model. This could indicate that the parameter values we used may be slightly off, e.g. up to about twofold; this is in fact consistent with the expected error range of our measurements (Cheikh et al., 2023). Alternatively, this could indicate that there are additional contributing factors that help allow ventral furrow formation to proceed normally in *scra* RNAi mutants. One potential factor might be cell rearrangements, which we do not include in our model. Allowing for cell rearrangements could possibly result in closer agreement between data and theory. Although we believe such corrections could yield an even more realistic model, we believe that the close match between experimental results and our 3D model strongly suggest that shear stresses play a major role in ventral furrow morphogenesis.

We would like emphasize that our simulation results qualitatively match several features seen *in vivo*. Most obviously, the time-evolution of cell shape changes in our simulations (Fig. 4) is qualitatively similar to those *in vivo* (Fig. 1) (also see Fig. S10 for a graphical comparison). In addition, as noted before, the membranes in the peripheral regions of the VF are longer than the central regions in the control, while the opposite is true in *scra* RNAi embryos *in vivo*. This observation is also seen in our simulations (Fig. S4). Additionally, the cytoplasmic flows previously characterized *in vivo* show good qualitative agreement with the corresponding data from our simulations (Fig. S9B), with both exhibiting a characteristic pattern of recirculation.

As an additional note, our time ramping computational experiments provided further evidence to that already published (Cheikh et al., 2023) that ventral furrow formation is an adiabatic process. In particular, we found that simulations in which active force is applied to the tissue instantaneously instead of being ramped up gradually, the simulated tissue invagination completes in less than 30 s (Fig. 4D). This result demonstrates that the time required by tissue invagination is limited by how quickly active forces build up, and not by the time required by the system to respond to those active forces.

## DISCUSSION

In this work, we explored VF formation in *scra* RNAi embryos, in which basal membranes do not form. Invagination proceeded normally, which was surprising in light of previous computational models positing that cellular volume conservation is required for VF invagination (Gelbart et al., 2012; Rauzi et al., 2013). Using both a 2D toy model and a 3D model with experimentally derived parameters, we explored the physical mechanisms underlying this surprising result. This analysis provides strong evidence that viscous shear is a key mechanical effect contributing to VF morphogenesis. In other words, VF formation may be considered a ‘swimming phenomenon’ where solid structures (cellular membranes) move by exerting force against the ambient fluid. A similar mechanism was previously proposed by Pouille and Farge (2008), although there the authors did not consider elasticity of cellular membranes, whereas elastic effects play a key role in ventral furrow formation, as was more recently demonstrated by Doubrovinski et al. (2018).

Previously published analyses of VF formation indicate that mesodermal cell elongation precedes invagination (Sweeton et al., 1991), so these are distinct processes. Our analysis here shows that cells elongate even in *scra* RNAi embryos that do not have basal membranes at all (Fig. 1H). Therefore, our results show that cell elongation, like the later process of invagination, does not require pressure difference across the basal membranes.

Importantly, our computational model indicates that basal membranes and viscous shear forces play complementary mechanical roles in wild-type embryos, since either is sufficient for these morphogenetic behaviors. In this particular system, the importance of viscous shear forces was revealed only in the *scra* RNAi background. However, there are many tissues in which similar effects might play the primary role. In the housefly *Musca vicina*, for example, it has been reported that gastrulation proceeds while the blastoderm is still syncytial (Bhuiyan and Shafiq, 1959). Additionally, phenomena involving viscous flows have been unexpectedly implicated in *Drosophila* oocyte growth (Lu et al., 2022) and Kupffer's vesicle development in zebrafish (Erdemci-Tandogan et al., 2018). Further afield evolutionarily, we note that, for example, the slime mold *Physarum polycephalum* (Alim et al., 2017) and the glass sponges (class *Hexactinellida*) (Leys et al., 2006) generate complex 3D shapes as syncytial organisms, suggesting that shear forces should also be investigated in these model systems.

## MATERIALS AND METHODS

### *Drosophila* genetics

For anillin depletion experiments (Figs 1 and 2), we used the following genotype: UAS-Nrt-eGFP; TRiP.GL01269^attP2^/mat15-GAL4. The TRiP.GL01269 was derived from RRID:BDSC 41841 (Bloomington *Drosophila* Stock Center); mat15-GAL4 was derived from RRID:BDS_80361 (Bloomington *Drosophila* Stock Center). To generate UAS-Nrt-eGFP, full-length Nrt-RB was cloned by PCR from LD22004 (*Drosophila* Genomics Resource Center stock 5736) and inserted into pPWG (*Drosophila* Genomics Resource Center stock 1078). This plasmid was injected by BestGene using P-element insertion. Control embryos were the same genotype as for anillin depletion, but included TRiP.HMS05302^attP40^ (expressing Loxl1 RNAi; from Bloomington *Drosophila* Stock Center RRID:BDSC 63028) instead of TRiP.GL01269^attP2^ in Fig. S2C,D, and simply omitting TRiP.GL01269^attP2^ for all other experiments.

### Fluorescent immunohistochemistry

Embryos were collected from female progeny on grape agar plates supplemented with yeast paste after at least 3.5 h. Fly embryos were heat-methanol fixed as described previously (Müller and Wieschaus, 1996). The block, primary antibody and secondary antibody steps were all carried out overnight with nutation at 4°C. Primary and secondary antibodies were diluted in block solution (1×PBS, 0.1% Triton X-100 and 5% heat inactivated goat serum or 0.2% BSA). Antibodies used include mouse anti-Neurotactin (1:50) (DSHB, BP 106, anti-Neurotactin, RRID:AB_528404), guinea pig anti-Snail (1:2000) (a gift from Eric Wieschaus, Princeton University, NJ, USA), goat anti-mouse IgG-Alexa Fluor 488 (1:500) (Invitrogen, A-11001) and goat anti-guinea pig IgG-Alexa Fluor 568 (1:500) (Invitrogen, A-11004). Nuclear staining was carried out using DAPI (1 μg/ml) (Invitrogen).

### Confocal fluorescence imaging of embryo sections

Immunostained fly embryos were staged in 1×PBS with 0.1% Triton X-100 under bright field on an Accu-Scope dissection microscope. A coverslip was prepared by cutting off ∼1 cm of the short edge and placing it in the center of the coverslip, perpendicular to the long edge, then adding a linear pool of AquaPolymount along the supporting glass strip. Selected gastrulas were transferred to the pool of mounting medium and sectioned along the dorsal-ventral axis using a 22 g needle. Embryo halves were positioned cut side down, leaning against the supporting glass strip. *Z*-stacks were imaged using a Plan-Apochromat 63×/1.40 oil objective on a Zeiss LSM 700 confocal microscope. For immunohistochemistry experiments, we fixed and stained samples over the course of several weeks, until we had at least 40 separate gastrulas imaged. From these, we selected all samples in which the morphology had not been significantly distorted by sectioning damage or angle – these results are quantified in the Fig. 1 bar graphs.

### Statistical analysis of immunostaining results

For the purposes of all experiments, all samples are biological replicates. We binned data from bar charts in Fig. 1 (maximum number of 20 positions, −10 to 9) in sequential groups of three, producing a plot with six average values of membrane lengths along the VF. We then compared experimental groups pairwise using Fisher's linear discriminant analysis. Specifically, we produced two sets of projections on the direction normal to the discriminant hyperplane, each set corresponding to one of the experimental groups being compared. Finally, the significance of the difference between the two sets of projections was assayed using the two-sample Kolmogorov–Smirnov test. For Figs S4 and S5, all data were used in analysis without any binning.

### Transmission electron microscopy

Briefly, we froze gastrulas of various stages under high pressure, subjected them to freeze substitution, made 60-70 nm transverse cross-sections and imaged them under TEM. A combined high pressure freezing and freeze substitution (HPF/FS) method was used to fix fly embryos as described elsewhere (Zhang and Chen, 2008). A Wohlwend Compact 03 high pressure freezer was used to fix the embryos. Samples were freeze substituted in 1% osmium tetroxide, 0.1% uranyl acetate in 98% acetone and 1% methanol using a Leica EM AFS2. The embryos were embedded in Embed-812 resin and polymerized in a 60°C oven overnight. Blocks were sectioned with a diamond knife (Diatome) on a Leica Ultracut 7 ultramicrotome (Leica Microsystems) and collected onto slot grids and post-stained with 2% aqueous uranyl acetate and lead citrate. Images were acquired on a JEOL JEM-1400 Plus TEM equipped with a LaB_6_ source operated at 120 kV using an AMT-BioSprint 16 M CCD camera. For TEM experiments, we continuously staged and fixed embryos over the course of 6-9 h. Embryo sections shown in Fig. 2 are representative of 5 (Fig. 2A), 3 (Fig. 2B), 11 (Fig. 2C) or 4 (Fig. 2D) samples. From these, we selected all samples in which the morphology had not been significantly distorted by ice and/or mechanical damage or other staining artifacts.

### Mechanical measurements of cytoplasm properties

Using a fire-polished holding pipette, we introduced individual magnetic microspheres (US Research Nanomaterials, US1163M) into the yolk sack of a developing embryo, and positioned them close to the basal hole of one of the cells. Next, using externally applied magnetic force, we pulled the microsphere into the cell through its basal opening. Finally, again using a magnet, we pulled the microsphere until it reached the apical surface, then removed the magnetic field. Analysis of the trajectory of the microsphere during and after pulling was carried out in MatLab.

### Mathematical modeling

Technical details of model implementation are described in the supplementary Materials and Methods. The code used to generate the results is publicly available at https://github.com/doubrovinskilab/anillin_code. However, for completeness of our presentation, we give a brief non-technical account of our modeling approach here. Our simulation techniques are standard, combining the Finite Element Method for simulating the fluid with the Immersed Boundary Method to consistently couple the dynamics of the fluid with the dynamics of the solid cellular boundaries. Note additionally that our modeling strategy is the same as was used in our previous work to develop a comprehensive mechanical model of embryonic epithelium (Cheikh et al., 2023).

Our model essentially describes the embryo as a network of cell membranes (approximated as elastic plates) immersed in liquid (the cytoplasm/yolk inside and the perivitelline fluid outside). To mimic the activity of myosin contraction, the membranes are subjected to contractile stress (Fig. S9A). To calculate the resulting course of deformation, one must specify the material properties of the membranes and of the liquid. For simplicity, we can assume that the membranes are linearly elastic. This amounts to assuming that the elastic reaction force in each membrane is linearly related to its deformation, which is merely a generalization of the familiar force law for the Hookean spring. The corresponding equations of linear elasticity involve two parameters: Young's modulus (which is the 3D counterpart of the spring constant measuring the force required to stretch the material a given amount) and Poisson's ratio (quantifying how much a material shrinks in one direction, when it is being stretched along the perpendicular direction). Again, for simplicity, we will assume that that fluid surrounding the membranes is described by Stokes equations, which is the simplest possible model of a viscous fluid. Stokes equations involve a single parameter, fluid viscosity. Finally, it is well known that when a solid is moving in a liquid, fluid velocity at the surface of the solid is the same as the velocity of the solid (the no slip boundary condition). Crucially, once the material properties of the membranes (Young's modulus and Poisson's ratio) and of its surrounding fluid (viscosity) are chosen, the dynamics of the system are determined uniquely from the instantaneous configuration of the system and the distribution of the active force driving the deformation. The no slip boundary condition uniquely determines how the solid and the fluid are coupled mechanically.

To simulate the physics described above, we use the Immersed Boundary Method, which we describe very briefly here. First, one subdivides the fluid into a discrete set of points (or nodes) with the three components (x, y, z) of fluid velocities specified at each one of those nodes. Likewise, the membranes are discretized as a triangulated mesh of connected solid nodes (surrounded by fluid nodes). A convenient description of the solid involves representing that solid as a network of Hookean springs (Seung and Nelson, 1988). Within the Immersed Boundary Method, each simulation time-step involves the following five computational steps.

(1) First, one determines the force acting at every node of the solid mesh. This force has two contributions: elastic force in the solid calculated as the sum of forces from all springs adjacent to a given node, and (possibly) an active force contribution that causes the solid to deform.

(2) The ‘spreading’ step. In this step, forces from solid nodes are transferred to adjacent fluid nodes. In its simplest form, this involves applying the force calculated at a given solid node to its closest fluid node.

(3) Next, fluid velocities at all the fluid nodes are calculated from the now known forces acting on the fluid by solving (a discretized approximation of) Stokes equation.

(4) The ‘interpolation’ step. The now known velocities at the fluid nodes are used to calculate velocities at the solid nodes. Since the velocity of the fluid at the fluid-solid boundary is the same as that of the solid, this may be achieved by approximating the velocity of a solid node as that of the closest fluid node.

(5) Finally, each solid node is translated for some short interval of time with the velocity of that node. This results in a new configuration of the solid and the procedure is repeated.

By iterating steps 1-5, one may determine the time-course of the deformation for any desired period of time.

It is worth pointing out that for a given spatial pattern of force, there is a unique pattern of velocities at small Reynold's numbers, which is the parameter regime in which both the experiments and models operate. That is, when any fluid moves sufficiently slowly, inertia is negligible, and fluid motion is determined by instantaneous force alone, and not by the previous history of the flow. The influence of inertia can be quantified by calculating the Reynold's number characterizing the flow. In the case of tissue movement during *Drosophila* gastrulation, Reynolds number has a value of ∼10^−8^ (Cheikh et al., 2023), which means that viscous forces exceed inertial forces by eight orders of magnitude. In this case, the motion of the cytoplasm is determined entirely by the (instantaneous magnitude of the) forces that it is subjected to. Since the parameters we use in our model come directly from *in vivo* measurements, the same thing applies.

## Supplementary Material



10.1242/develop.202892_sup1 Supplementary information

## References

[DEV202892C1] Alim, K., Andrew, N., Pringle, A. and Brenner, M. P. (2017). Mechanism of signal propagation in Physarum polycephalum. *Proc. Natl. Acad. Sci. USA* 114, 5136-5141. 10.1073/pnas.1618114114 28465441 PMC5441820

[DEV202892C2] Barrett, K., Leptin, M. and Settleman, J. (1997). The Rho GTPase and a putative RhoGEF mediate a signaling pathway for the cell shape changes in Drosophila gastrulation. *Cell* 91, 905-915. 10.1016/S0092-8674(00)80482-1 9428514

[DEV202892C3] Bhuiyan, N. I. and Shafiq, S. A. (1959). The differentiation of the posterior pole-plasm in the housefly Musca vicina Macquart. *Exp. Cell Res.* 16, 427-429. 10.1016/0014-4827(59)90274-5 13653006

[DEV202892C4] Campos-Ortega, J. A. and Hartenstein, V. (1985). *The Embryonic Development of Drosophila melanogaster*. Berlin: Springer.

[DEV202892C5] Cheikh, M. I., Tchoufag, J., Osterfield, M., Dean, K., Bhaduri, S., Zhang, C., Mandadapu, K. K. and Doubrovinski, K. (2023). A comprehensive model of Drosophila epithelium reveals the role of embryo geometry and cell topology in mechanical responses. *eLife* 12, e85569. 10.7554/eLife.85569.sa2 37782009 PMC10584372

[DEV202892C6] Costa, M., Wilson, E. T. and Wieschaus, E. (1994). A putative cell signal encoded by the folded gastrulation gene coordinates cell shape changes during Drosophila gastrulation. *Cell* 76, 1075-1089. 10.1016/0092-8674(94)90384-0 8137424

[DEV202892C7] Dawes-Hoang, R. E., Parmar, K. M., Christiansen, A. E., Phelps, C. B., Brand, A. H. and Wieschaus, E. F. (2005). folded gastrulation, cell shape change and the control of myosin localization. *Development* 132, 4165-4178. 10.1242/dev.01938 16123312

[DEV202892C8] Doubrovinski, K., Swan, M., Polyakov, O. and Wieschaus, E. F. (2017). Measurement of cortical elasticity in Drosophila melanogaster embryos using ferrofluids. *Proc. Natl. Acad. Sci. USA* 114, 1051-1056. 10.1073/pnas.1616659114 28096360 PMC5293093

[DEV202892C9] Doubrovinski, K., Tchoufag, J. and Mandadapu, K. (2018). A simplified mechanism for anisotropic constriction in Drosophila mesoderm. *Development* 145, dev167387. 10.1242/dev.167387 30401702 PMC6307890

[DEV202892C10] Erdemci-Tandogan, G., Clark, M. J., Amack, J. D. and Manning, M. L. (2018). Tissue flow induces cell shape changes during organogenesis. *Biophys. J.* 115, 2259-2270. 10.1016/j.bpj.2018.10.028 30455043 PMC6289824

[DEV202892C11] Field, C. M., Coughlin, M., Doberstein, S., Marty, T. and Sullivan, W. (2005). Characterization of anillin mutants reveals essential roles in septin localization and plasma membrane integrity. *Development* 132, 2849-2860. 10.1242/dev.01843 15930114

[DEV202892C12] Fierling, J., John, A., Delorme, B., Torzynski, A., Blanchard, G., Lye, C., Malandain, G., Sanson, B., Etienne, J., Marmottant, P. et al. (2022). Embryo-scale epithelial buckling forms a propagating furrow that initiates gastrulation. *Nat. Commun.* 13, 3348. 10.1038/s41467-022-30493-3 35688832 PMC9187723

[DEV202892C13] Gelbart, M. A., He, B., Martin, A. C., Thiberge, S. Y., Wieschaus, E. F. and Kaschube, M. (2012). Volume conservation principle involved in cell lengthening and nucleus movement during tissue morphogenesis. *Proc. Natl. Acad. Sci. USA* 109, 19298-19303. 10.1073/pnas.1205258109 23134725 PMC3511084

[DEV202892C14] Gracia, M., Theis, S., Proag, A., Gay, G., Benassayag, C. and Suzanne, M. (2019). Mechanical impact of epithelial-mesenchymal transition on epithelial morphogenesis in Drosophila. *Nat. Commun.* 10, 2951. 10.1038/s41467-019-10720-0 31273212 PMC6609679

[DEV202892C15] He, B., Doubrovinski, K., Polyakov, O. and Wieschaus, E. (2014). Apical constriction drives tissue-scale hydrodynamic flow to mediate cell elongation. *Nature* 508, 392-396. 10.1038/nature13070 24590071 PMC4111109

[DEV202892C16] Heer, N. C., Miller, P. W., Chanet, S., Stoop, N., Dunkel, J. and Martin, A. C. (2017). Actomyosin-based tissue folding requires a multicellular myosin gradient. *Development* 144, 1876-1886. 10.1242/dev.146761 28432215 PMC5450837

[DEV202892C17] Izquierdo, E., Quinkler, T. and De Renzis, S. (2018). Guided morphogenesis through optogenetic activation of Rho signalling during early Drosophila embryogenesis. *Nat. Commun.* 9, 2366. 10.1038/s41467-018-04754-z 29915285 PMC6006163

[DEV202892C18] Kam, Z., Minden, J. S., Agard, D. A., Sedat, J. W. and Leptin, M. (1991). Drosophila gastrulation: analysis of cell shape changes in living embryos by three-dimensional fluorescence microscopy. *Development* 112, 365-370. 10.1242/dev.112.2.365 1794308

[DEV202892C19] Kanesaki, T., Hirose, S., Grosshans, J. and Fuse, N. (2013). Heterotrimeric G protein signaling governs the cortical stability during apical constriction in Drosophila gastrulation. *Mech. Dev.* 130, 132-142. 10.1016/j.mod.2012.10.001 23085574

[DEV202892C20] Kerridge, S., Munjal, A., Philippe, J.-M., Jha, A., De Las Bayonas, A. G., Saurin, A. J. and Lecuit, T. (2016). Modular activation of Rho1 by GPCR signalling imparts polarized myosin II activation during morphogenesis. *Nat. Cell Biol.* 18, 261-270. 10.1038/ncb3302 26780298

[DEV202892C21] Kim, H. Y., Varner, V. D. and Nelson, C. M. (2013). Apical constriction initiates new bud formation during monopodial branching of the embryonic chicken lung. *Development* 140, 3146-3155. 10.1242/dev.093682 23824575 PMC3931740

[DEV202892C22] Kohler, R. E. (1994). *Lords of the Fly: Drosophila Genetics and the Experimental Life*, 1st edn. University of Chicago Press.

[DEV202892C23] Kölsch, V., Seher, T., Fernandez-Ballester, G. J., Serrano, L. and Leptin, M. (2007). Control of Drosophila gastrulation by apical localization of adherens junctions and RhoGEF2. *Science* 315, 384-386. 10.1126/science.1134833 17234948

[DEV202892C24] Krueger, D., Quinkler, T., Mortensen, S. A., Sachse, C. and De Renzis, S. (2019). Cross-linker-mediated regulation of actin network organization controls tissue morphogenesis. *J. Cell Biol.* 218, 2743-2761. 10.1083/jcb.201811127 31253650 PMC6683744

[DEV202892C25] Lecuit, T. and Wieschaus, E. (2000). Polarized insertion of new membrane from a cytoplasmic reservoir during cleavage of the Drosophila embryo. *J. Cell Biol.* 150, 849-860. 10.1083/jcb.150.4.849 10953008 PMC2175274

[DEV202892C26] Leptin, M. (1991). Mechanics and genetics of cell shape changes during Drosophila ventral furrow formation. In *Gastrulation: Movements Patterns and Molecules*, 1st edn (ed. R. Keller, W. H. Clark and F. Griffin), pp. 199-212. Springer.

[DEV202892C27] Leptin, M. and Grunewald, B. (1990). Cell shape changes during gastrulation in Drosophila. *Development* 110, 73-84. 10.1242/dev.110.1.73 2081472

[DEV202892C52] Leptin M., Casal J., Grunewald B. and Reuter R. (1992). Mechanisms of early Drosophila mesoderm formation. *Development*. 116, 23-31. doi:10.1242/dev.116.Supplement.23 1299365

[DEV202892C28] Leys, S. P., Cheung, E. and Boury-Esnault, N. (2006). Embryogenesis in the glass sponge Oopsacas minuta: Formation of syncytia by fusion of blastomeres. *Integr. Comp. Biol.* 46, 104-117. 10.1093/icb/icj016 21672727

[DEV202892C29] Loncar, D. and Singer, S. J. (1995). Cell membrane formation during the cellularization of the syncytial blastoderm of Drosophila. *Proc. Natl. Acad. Sci. USA* 92, 2199-2203. 10.1073/pnas.92.6.2199 7892247 PMC42451

[DEV202892C30] Lu, W., Lakonishok, M., Serpinskaya, A. S. and Gelfand, V. I. (2022). A novel mechanism of bulk cytoplasmic transport by cortical dynein in Drosophila ovary. *eLife* 11, e75538. 10.7554/eLife.75538 35170428 PMC8896832

[DEV202892C31] Lye, C. M. and Sanson, B. (2011). Tension and epithelial morphogenesis in Drosophila early embryos. *Curr. Top. Dev. Biol.* 95, 145-187. 10.1016/B978-0-12-385065-2.00005-0 21501751

[DEV202892C32] Manning, A. J., Peters, K. A., Peifer, M. and Rogers, S. L. (2013). Regulation of epithelial morphogenesis by the G protein-coupled receptor mist and its ligand fog. *Sci. Signal.* 6, ra98. 10.1126/scisignal.2004427 24222713 PMC4154316

[DEV202892C33] Martin, A. C., Kaschube, M. and Wieschaus, E. F. (2009). Pulsed contractions of an actin-myosin network drive apical constriction. *Nature* 457, 495-499. 10.1038/nature07522 19029882 PMC2822715

[DEV202892C34] Martin, A. C., Gelbart, M., Fernandez-Gonzalez, R., Kaschube, M. and Wieschaus, E. F. (2010). Integration of contractile forces during tissue invagination. *J. Cell Biol.* 188, 735-749. 10.1083/jcb.200910099 20194639 PMC2835944

[DEV202892C35] Martinez Arias, A. and Stewart, A. (2003). Molecular principles of animal development. *Q Rev. Biol.* 78, 94.

[DEV202892C36] Maruyama, R. and Andrew, D. J. (2012). Drosophila as a model for epithelial tube formation. *Dev. Dyn.* 241, 119-135. 10.1002/dvdy.22775 22083894 PMC3922621

[DEV202892C37] Mazumdar, A. and Mazumdar, M. (2002). How one becomes many: blastoderm cellularization in Drosophila melanogaster. *BioEssays* 24, 1012-1022. 10.1002/bies.10184 12386932

[DEV202892C38] Müller, H. A. and Wieschaus, E. (1996). armadillo, bazooka, and stardust are critical for early stages in formation of the zonula adherens and maintenance of the polarized blastoderm epithelium in Drosophila. *J. Cell Biol.* 134, 149-163. 10.1083/jcb.134.1.149 8698811 PMC2120925

[DEV202892C39] Perez-Mockus, G., Mazouni, K., Roca, V., Corradi, G., Conte, V. and Schweisguth, F. (2017). Spatial regulation of contractility by Neuralized and Bearded during furrow invagination in Drosophila. *Nat. Commun.* 8, 1594. 10.1038/s41467-017-01482-8 29150614 PMC5693868

[DEV202892C40] Polyakov, O., He, B., Swan, M., Shaevitz, J. W., Kaschube, M. and Wieschaus, E. (2014). Passive mechanical forces control cell-shape change during Drosophila ventral furrow formation. *Biophys. J.* 107, 998-1010. 10.1016/j.bpj.2014.07.013 25140436 PMC4142243

[DEV202892C41] Pouille, P.-A. and Farge, E. (2008). Hydrodynamic simulation of multicellular embryo invagination. *Phys. Biol.* 5, 015005. 10.1088/1478-3975/5/1/015005 18403824

[DEV202892C42] Rauzi, M., Brezavšček, A. H., Ziherl, P. and Leptin, M. (2013). Physical models of mesoderm invagination in Drosophila embryo. *Biophys. J.* 105, 3-10. 10.1016/j.bpj.2013.05.039 23823218 PMC3699736

[DEV202892C43] Sawyer, J. M., Harrell, J. R., Shemer, G., Sullivan-Brown, J., Roh-Johnson, M. and Goldstein, B. (2010). Apical constriction: a cell shape change that can drive morphogenesis. *Dev. Biol.* 341, 5-19. 10.1016/j.ydbio.2009.09.009 19751720 PMC2875788

[DEV202892C44] Selvaggi, L., Pasakarnis, L., Brunner, D. and Aegerter, C. M. (2018). Magnetic tweezers optimized to exert high forces over extended distances from the magnet in multicellular systems. *Rev. Sci. Instrum.* 89, 045106. 10.1063/1.5010788 29716356

[DEV202892C45] Seung, H. S. and Nelson, D. R. (1988). Defects in flexible membranes with crystalline order. *Physical Review A* 38, 1005-1018. 10.1103/PhysRevA.38.1005 9900464

[DEV202892C46] Sweeton, D., Parks, S., Costa, M. and Wieschaus, E. (1991). Gastrulation in Drosophila: the formation of the ventral furrow and posterior midgut invaginations. *Development* 112, 775-789. 10.1242/dev.112.3.775 1935689

[DEV202892C47] Thomas, J. H. and Wieschaus, E. (2004). src64 and tec29 are required for microfilament contraction during Drosophila cellularization. *Development* 131, 863-871. 10.1242/dev.00989 14736750

[DEV202892C48] Turner, F. R. and Mahowald, A. P. (1977). Scanning electron microscopy of Drosophila melanogaster embryogenesis. II. Gastrulation and segmentation. *Dev. Biol.* 57, 403-416. 10.1016/0012-1606(77)90225-1 406152

[DEV202892C49] Xue, Z. and Sokac, A. M. (2016). -Back-to-back mechanisms drive actomyosin ring closure during Drosophila embryo cleavage. *J. Cell Biol.* 215, 335-344. 10.1083/jcb.201608025 27799369 PMC5100295

[DEV202892C50] Zalokar, M. and Erk, I. (1976). Division and migration of nuclei during early embryogenesis of Drosophila melanogaster. *J. Microsc. Biol. Cell* 25, 97-106.

[DEV202892C51] Zhang, S. and Chen, E. H. (2008). Ultrastructural analysis of myoblast fusion in Drosophila. *Methods Mol. Biol.* 475, 275-297. 10.1007/978-1-59745-250-2_16 18979250 PMC4408380

