# Supplementary material for: Viscous shear is a key force in Drosophila ventral furrow morphogenesis
Source: Development. 2024 Nov 15;151(22):dev202892. doi: 10.1242/dev.202892 (PMC11586522; doi:10.1242/dev.202892)
Supplement: Supplementary information [file develop-151-202892-s1.pdf]

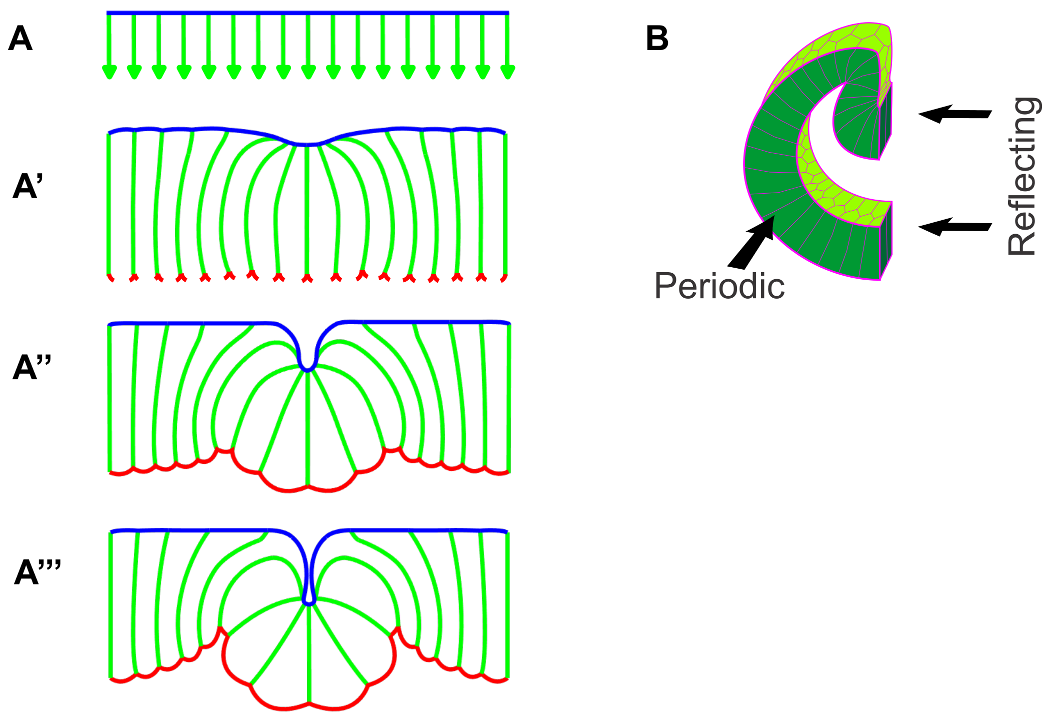

Fig. S1. Schematic of membrane shape changes throughout VF formation and the geometry of the model.

(A-A''') Illustration of VF formation in cross section showing shape changes in apical (blue), lateral (green), and basal (red) membranes throughout tissue folding. (A) Lateral membranes lengthen (arrows) throughout cellularization. (A') Lateral membranes of VF cells lengthen more as apical membranes constrict to initiate VF formation. (A"-A"') Lateral membranes shrink back down as tissue invaginates and basal membranes form. Basal membranes expand as the VF continues to fold inward.

(B) Schematic illustrating the geometry of the simulated domain and boundary conditions.

Development • Supplementary information

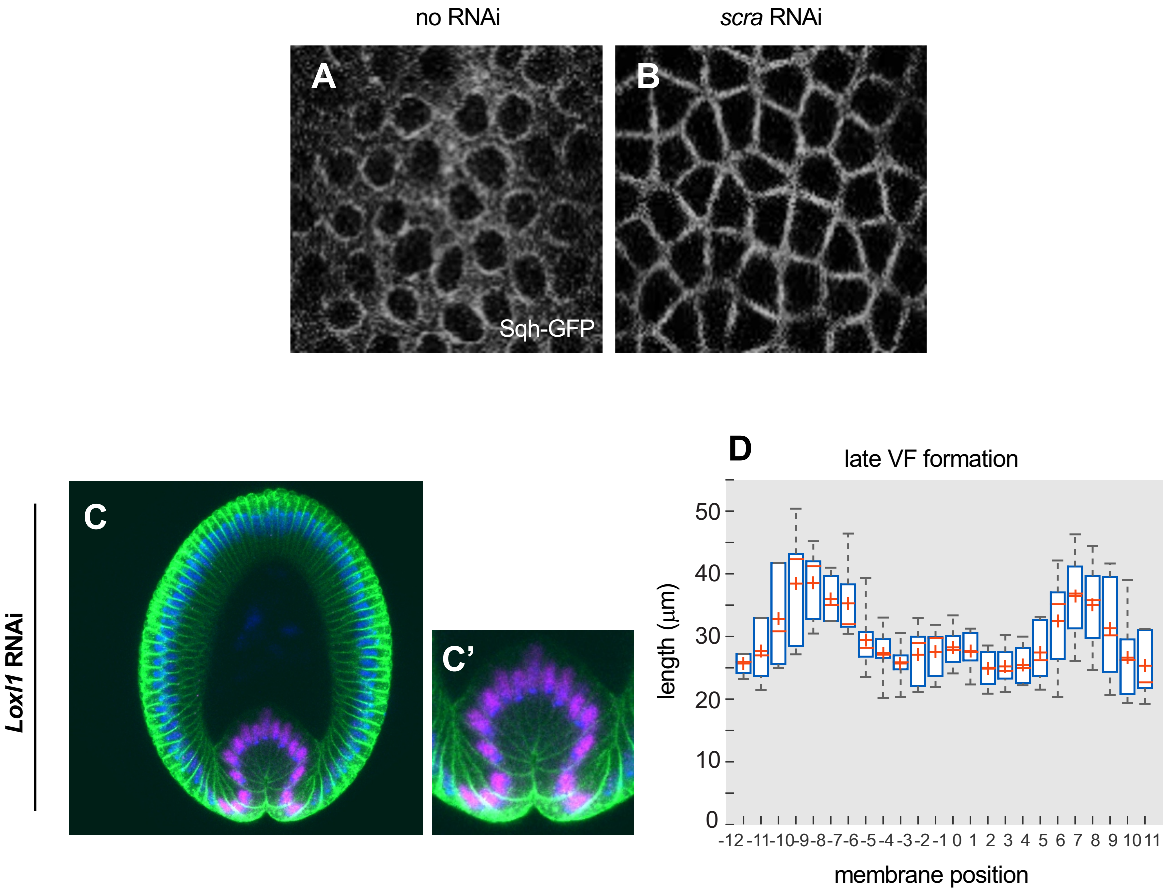

Fig. S2. Further characterization of *scra* RNAi and control embryos.

(A,B) Maximum intensity projections of the cellularizing ventral epithelium in live control (A) and *scra* RNAi (B) embryos during late cellularization. Acto-myosin rings remain open and are misshapen in *scra* RNAi embryos, preventing the formation of basal membranes.

(C-D) Confocal immunofluorescence (C-C'), and quantification of tissue morphology (D) in *Loxl1* RNAi control embryos. These controls match the genotype used for Figure 1G-L, except for including a short hairpin RNA against *Loxl1* instead of against *scra*.

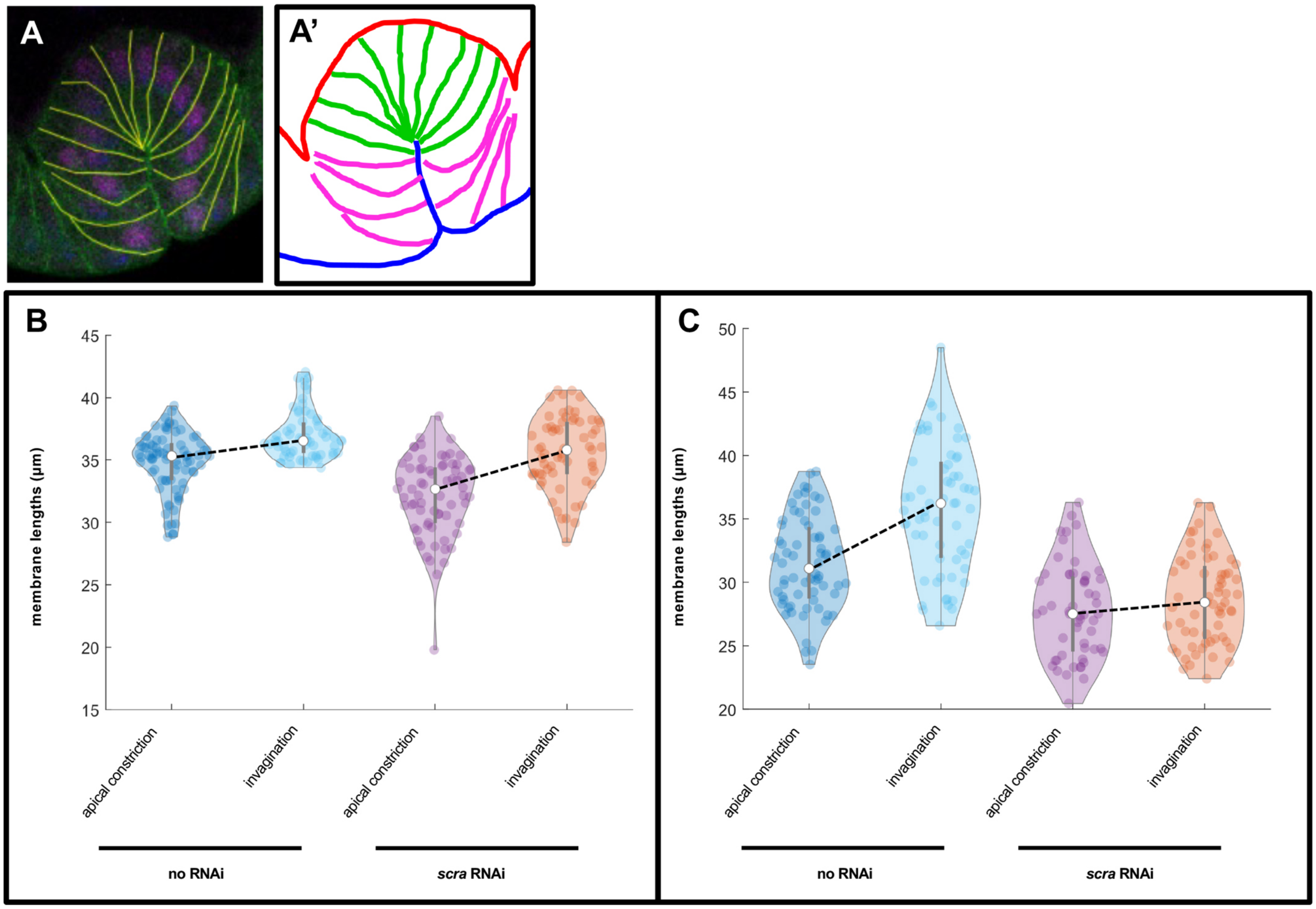

Fig. S3. Central mesodermal cells lengthen more, peripheral mesodermal cells shorten less throughout VF formation in *scra* RNAi embryos compared to wild-type.

Example of designated central (green) and peripheral (pink) mesoderm regions shown in (A,A'). Violin plots show changes in average central (B) and peripheral mesodermal membrane lengths (C) between apical constriction (ac) and invagination in wild-type (n_{ac} = 9 embryos, n_{invag} = 7 embryos) and scra RNAi embryos ($n_{ac} = n_{invag}$ = 8 embryos).

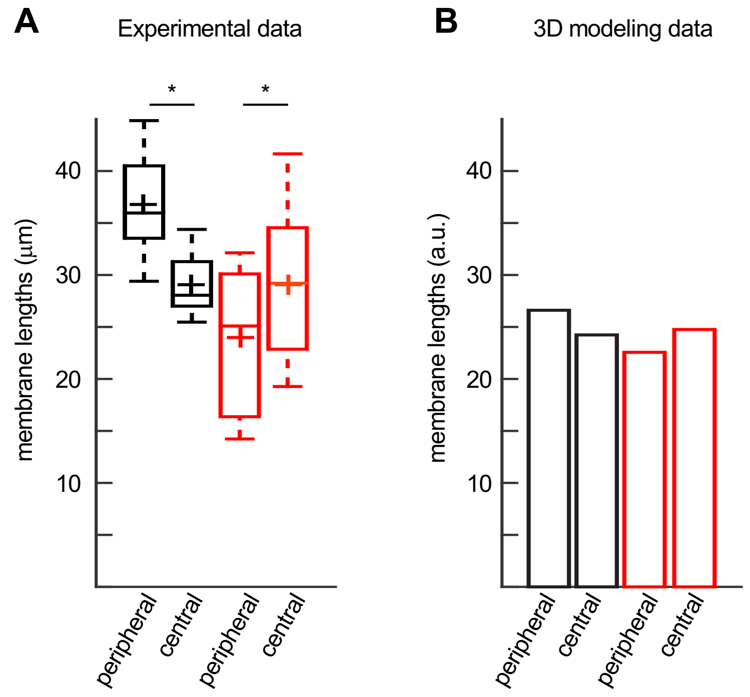

Fig. S4. Model accounts for *in vivo* membrane length differences between membranes at edge and center of mesoderm.

(A) Lengths of membranes in peripheral VF compared to central VF (see figure S3A) in no RNAi control (black, n=11 embryos) and scra RNAi (red, n = 9 embryos) embryos during late VF formation. Data are represented as box and whisker plots, where the central mark is the median, the cross is the mean, and the box edges are the 25th and 75th percentiles. Significance was determined by two-sample Kolmogorov-Smirnov test (* = $p < 0.05$). (B) Lengths of equivalently-located membranes in 3D model embryos at the final stage: control (black) and *scra* RNAi (red). In both experiment and model, peripheral membranes are longer than central membranes in the control, but this is reversed in *scra* RNAi.

Development • Supplementary information

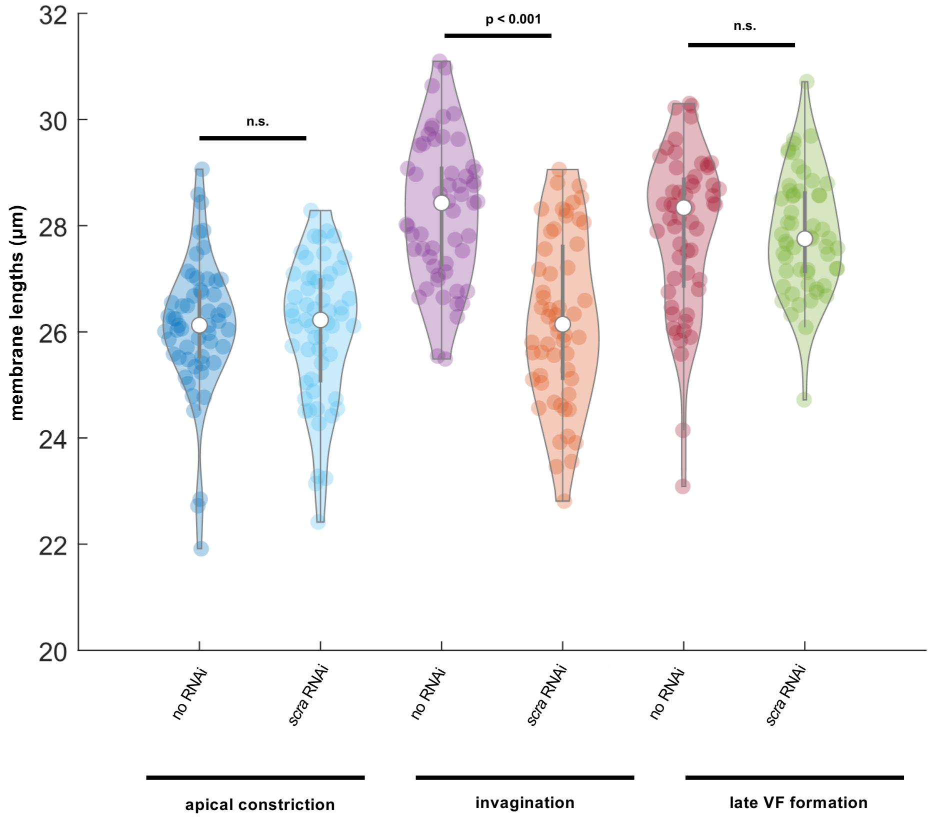

Fig. S5. Non-mesodermal cells are shorter during invagination in *scra* RNAi embryos.

Violin plots of non-mesodermal cell lengths during apical constriction, invagination, and late VF formation in wild-type (wt) and *scra* RNAi embryos. Non-mesodermal cells of *scra* RNAi embryos do not lengthen as quickly as those in control, resulting in shorter epithelium mid VF-formation. By late VF formation, non-mesodermal cells of *scra* RNAi embryos have reached lengths similar to those in wild-type embryos. Significance was determined by two-sample Kolmogorov-Smirnov test.

Development • Supplementary information

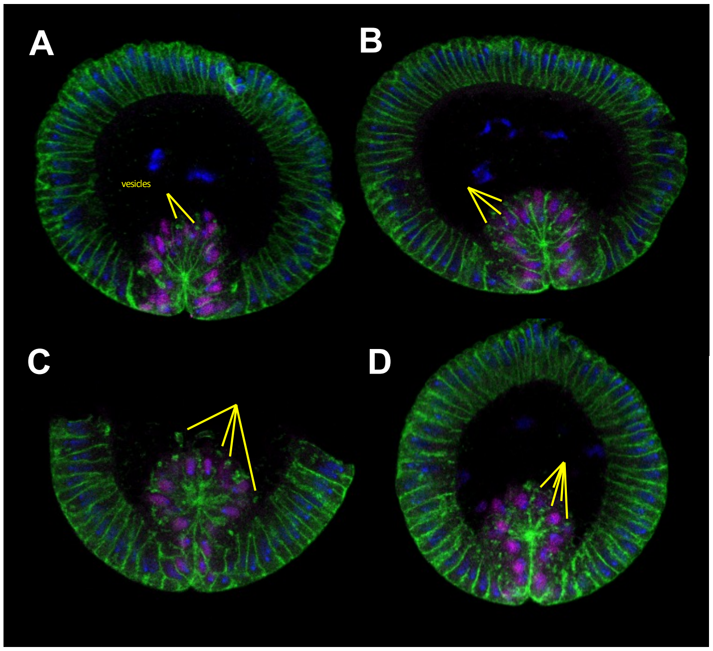

Fig. S6. Membranes in *scra* RNAi embryos deteriorate more over time.

Confocal immunofluorescence of heat/methanol-fixed anillin RNAi embryos at increasingly older stages of gastrulation (A-D). Membrane degradation increases with age, and vesicles become more numerous. Note: the embryo section in C is incomplete due to how it was cut, not any depleted protein expression or lack of antibody staining.

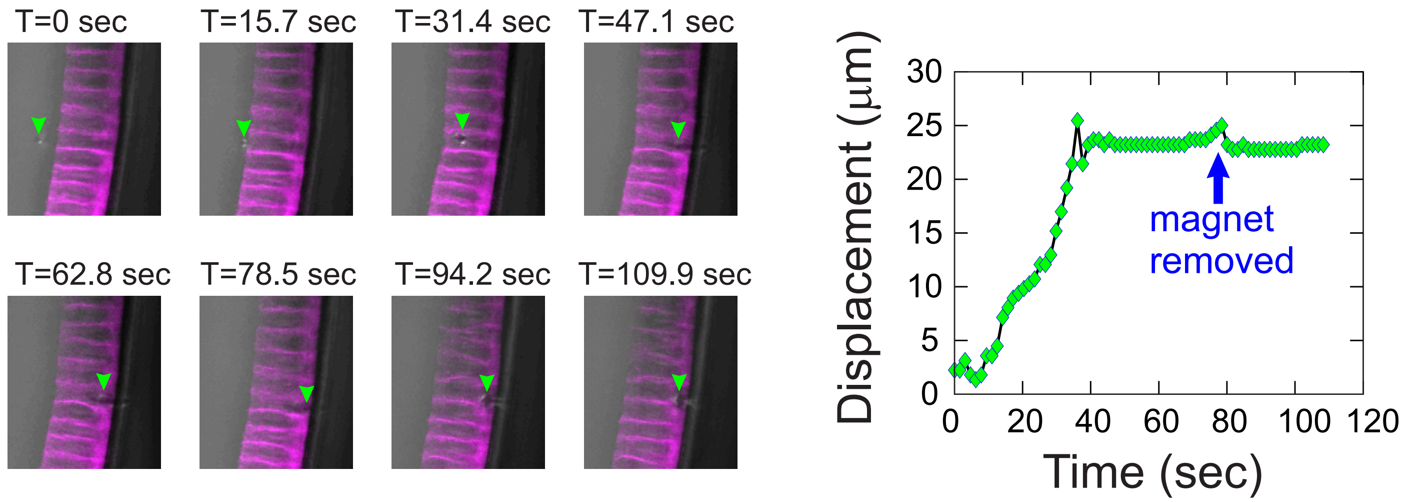

Fig. S7. Cellular interior is viscous.

Left: a sequence of snapshots, showing a magnetic bead pulled into a cell through the opening on the basal side, and subsequently pulled all the way to the apical surface. Green arrowheads mask the position of the bead. Cell membranes are labeled with CellMask dye injected into the perivitelline space. Right: tracked position of the bead. The time point when magnet was removed is marked with a blue arrow. Note complete lack of subsequent elastic recoil.

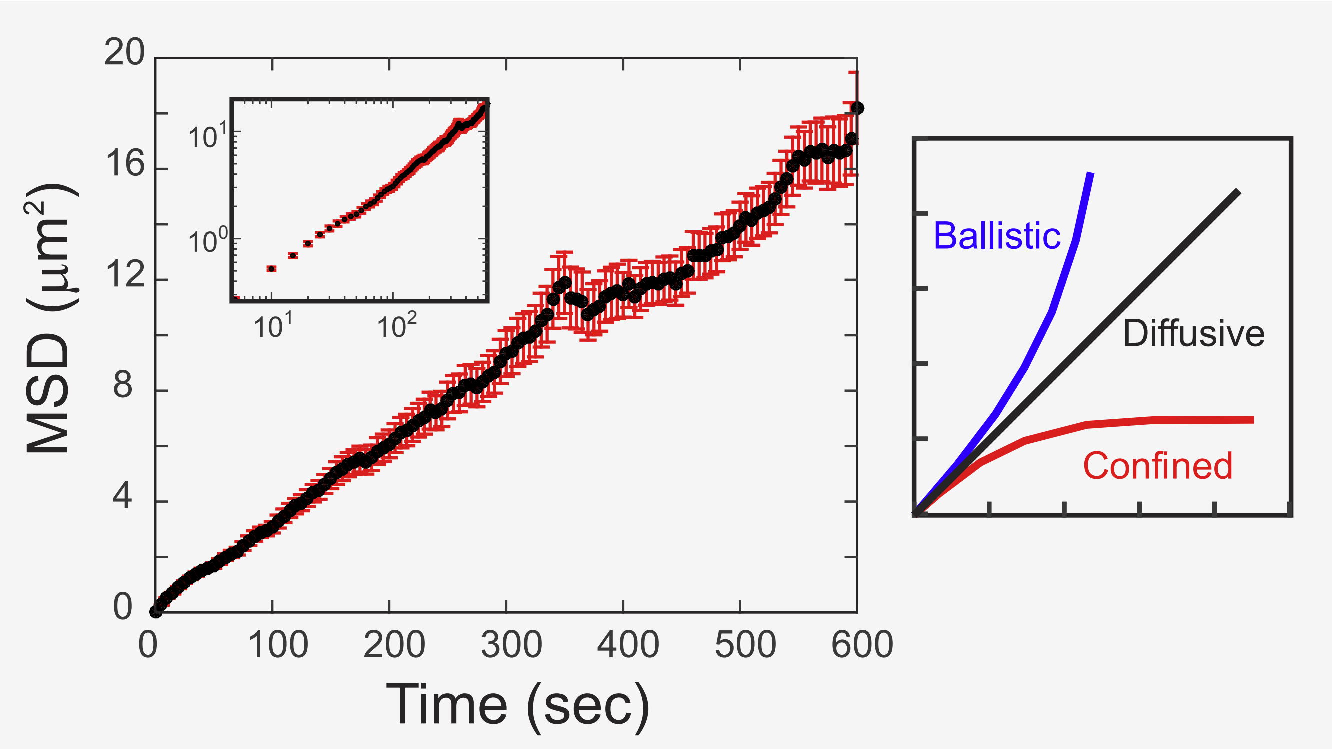

Fig. S8. The motion of 0.5 micron beads within the cytoplasm is diffusive.

Left: mean square displacement of 0.5 micron beads as a function of time; error bars show standard error of the mean. The data graphed are derived from 1,899 tracks from 5 cellular-stage embryos. The dependence is approximately linear indicating that bead motion is predominantly diffusive. Right: a schematic explaining the expected behavior of similar experiments in three regimes: confined (corresponding to diffusion in elastic media), ballistic (corresponding to unidirectional active transport) and diffusive (corresponding to diffusion in viscous media).

Development • Supplementary information

A

Tension distribution

Wild type	Apical	Lateral (apico-basal)	Basal
mesoderm	160	16	2
ectoderm	0.5	0.5	0.5

Anillin RNAi	Apical	Lateral (apico-basal)	Basal
mesoderm	160	16	-
ectoderm	0.5	0.5	-

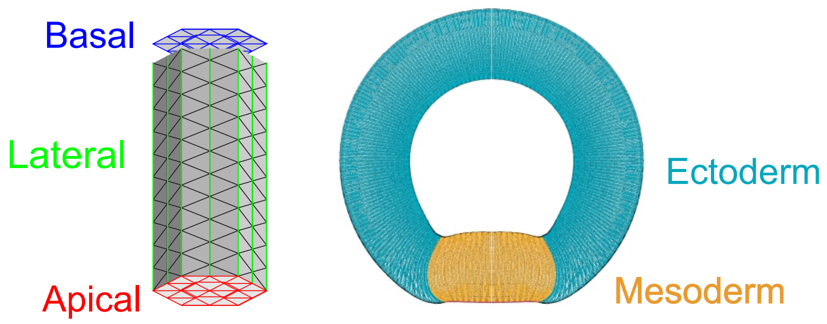

B

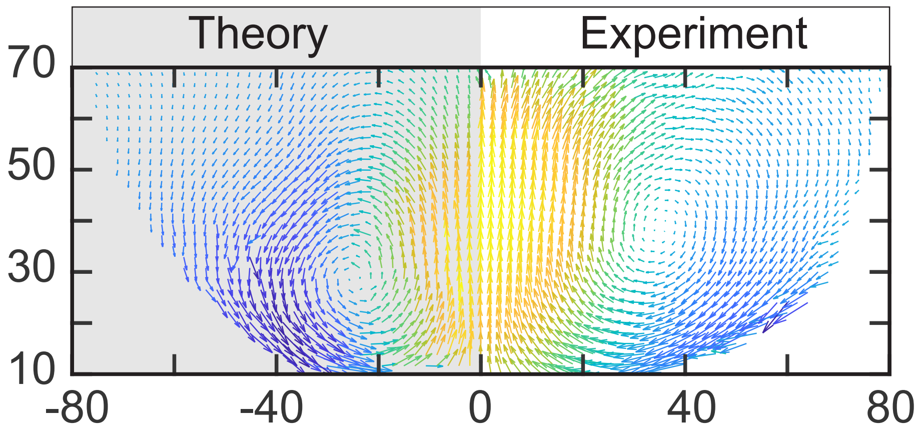

Fig. S9. Active stress and resulting cytoplasmic flows in the 3D model gastrula.

A) Distribution of active stresses in the different membrane domains, qualitatively based on previously reported myosin distribution (Dawes-Hoang et al., 2005, Gracia et al., 2019). Relative magnitudes of those stresses remain the same throughout the course of the simulation, whereas absolute magnitudes are ramped from zero to their final value. The values in the chart show tension assigned to each class of mesh elements, depending on cell type (mesoderm or ectoderm) and cellular domain (apical, lateral, or basal). More specifically, the magnitude of active stress (in units of nN/μm) at time t seconds is given by the value in the table multiplied by $10^{-5}t$. Note that stresses in the lateral domains are only assigned to edges directed along the apical-basal axis; the other mesh elements in the lateral domain are assigned a tension of zero. B) Patterns of cytoplasmic flows obtained from the model (left) and measured experimentally (right). Note the characteristic vortex of recirculation. The pattern on the left corresponds to an early stage in the simulation from Figure 4c, the pattern on the right is re-plotted from experimental shown in Figure 1D of our previous work (He et al., 2014).

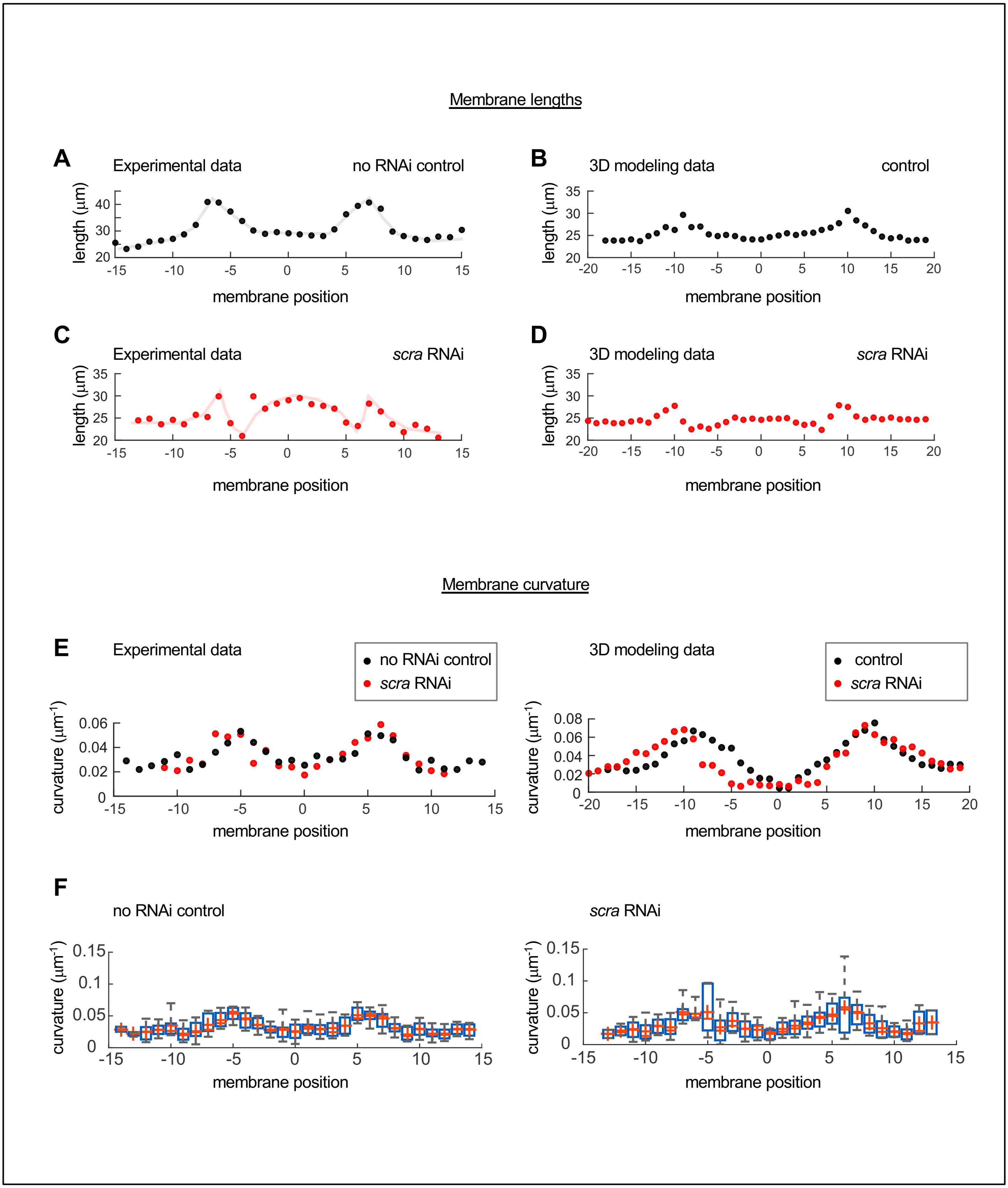

Development • Supplementary information

Fig. S10. Comparison of measurement data with modeling results.

A) Comparison of membrane lengths across different conditions, as indicated in the figure labels. Experimental results are from late VF formation stage; model results are from the final computed state. Experimental data shown here (A,C) are mean values; the box and whisker representation of the full data sets are in Figure 1F and 1L.

(E-F) Comparison of membrane curvatures across different conditions. Average curvature for each membrane (experimental or model-derived) was calculated as follows: Each membrane was discretized into line segments, and angles between consecutive line segments were calculated. The average curvature was approximated as the sum of the absolute values of the angles divided by the total length of the curve. The x-axis in each histogram is the position of a membrane counted as the number of cells between it and the ventral midline, with negative numbers to the left and positive to the right. For experimental data, the mean of 9 embryos (for *scra* RNAi) or the mean of 11 embryos (for control) is shown in the left panel of E. Box and whisker plots for the same experimental data are shown in F, where the central mark is the median, the cross is the mean, and the box edges are the 25th and 75th percentiles.

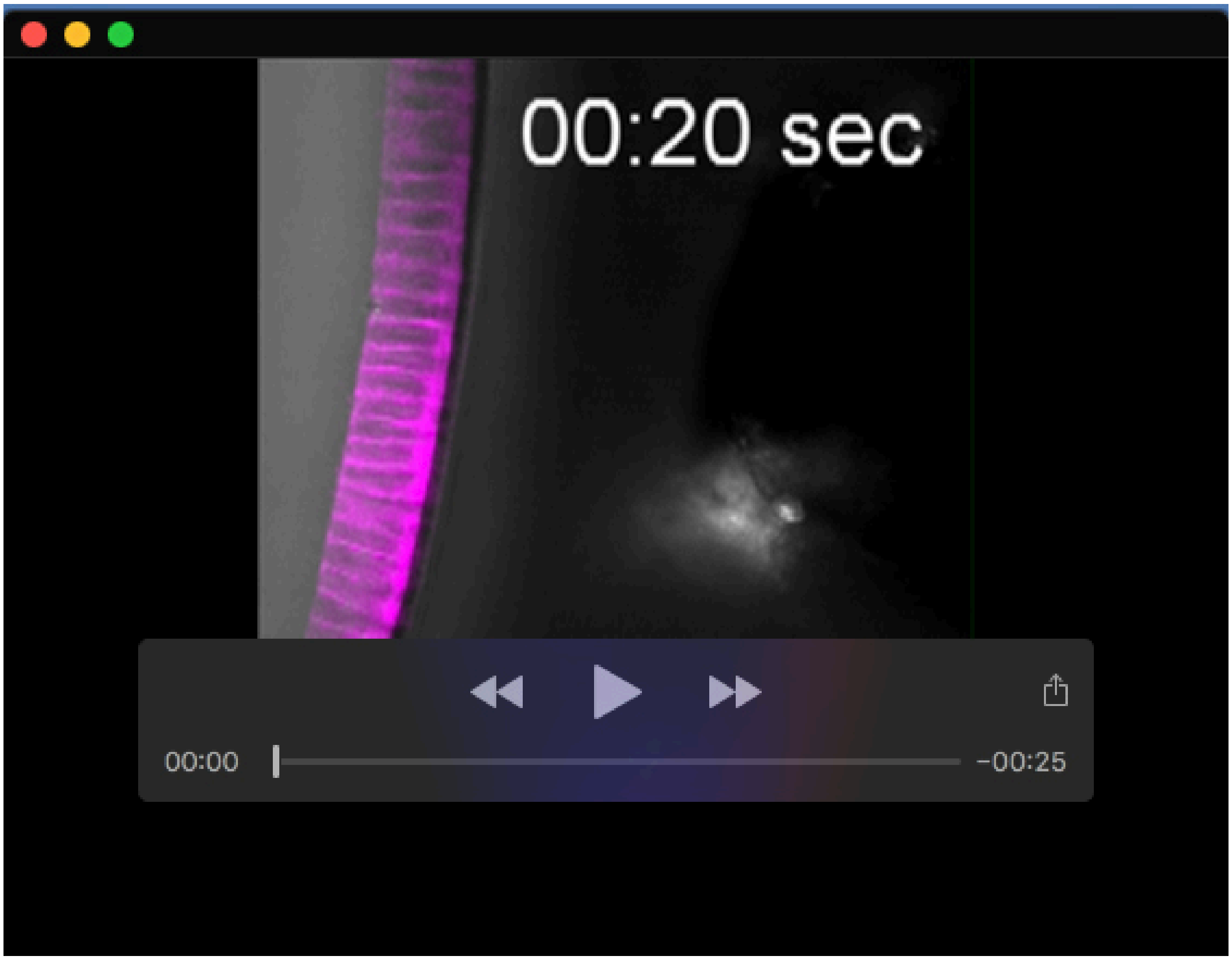

Movie 1. Cellular interior is viscous.

A video corresponding to the snapshots in Supplemental Figure S7, showing a magnetic bead (black dot, from bright field channel) pulled into a cell through the opening on the basal side, and subsequently pulled all the way to the apical surface. Cell membranes are labeled with CellMask dye injected into the perivitelline space. Note complete lack of subsequent elastic recoil.

SUPPLEMENTARY MATERIALS AND METHODS

Numerical simulations of ventral furrow formation

In this section, we detail the numerical method used to simulate ventral furrow formation. Our approach is a combination of the Immersed Boundary Method (IBM) (Peskin, 1972; Peskin and Printz, 1993) and the Finite Element Method (FEM) (Boffi et al., 2007). The choice of the numerical method is motivated by the requirement to describe fluid-structure interactions, since epithelial cells comprising gastrulation have previously been shown to be predominantly elastic on their surface and viscous in the interior (Cheikh et al., 2023; Doubrovinski et al., 2017; Rauzi et al., 2008). A key advantage of the IBM approach is that the computational (Eulerian) grid representing the fluid remains fixed throughout a simulation, while the solid (Lagrangian) mesh deforms. This approach avoids the need to re-mesh the fluid throughout the simulations. Technical details of our numerical implementation and validation of the method are given elsewhere (Cheikh et al., 2023).

Governing Equations

For both 2D and 3D simulations, time evolution of the fluid-immersed interfaces representing cellular edges requires simulating the ambient fluid, whose dynamics are given by the Stokes equations:

$$\nabla \cdot \boldsymbol{u}_f = 0, \quad (1)$$

$$-\nabla p_f + \mu \nabla^2 \boldsymbol{u}_f = \boldsymbol{f}_f. \quad (2)$$

Here μ is dynamic viscosity, while $\boldsymbol{u}_f(x,t)$ and $p_f(x,t)$ are the velocity and the pressure fields respectively. $\boldsymbol{f}_f$ is the force density acting on the fluid. Importantly, $\boldsymbol{f}_f$ includes the force on the fluid from the immersed structure. Note that the pressure term acts to impose volume conservation in each of the two fluid compartments, 1. the combined cell cytoplasm and yolk, and 2. the perivitelline space. We are justified in neglecting inertia since a typical Reynolds number in our problem may be estimated to be on the order of Re = 10^{-8} (Doubrovinski et al., 2017; Selvaggi et al., 2018). Cell edges are described as linearly elastic shells characterized by a (two-dimensional) Young's modulus E and Poisson's ratio σ. As detailed in (Seung and Nelson, 1988), it is convenient to approximate the immersed solid surfaces as a triangulated mesh of approximately equilateral triangular elements. Setting the spring constant of triangle element edges to k, in the continuum limit one recovers an approximation of a linearly elastic sheet with a Young's modulus of $E = \frac{2}{\sqrt{3}}k$ and a Poisson's ration of $\sigma = \frac{1}{3}$. The dynamics are driven by active compressive stresses (corresponding to the action of myosin motors) described as force dipoles of constant magnitude (i.e. independent of the length of an edge), directed along the edges of the triangular elements discretizing the solid. In this way, the ith solid edge contributes a force dipole to the $\boldsymbol{f}_f$-term on the right-hand side of equation (2):

$$\boldsymbol{f}_s = (k\Delta L + \gamma)\boldsymbol{n}, \quad (3)$$

where k is the elastic constant of a solid edge, ΔL is elongation distance, γ determines the magnitude of active stress, and $\boldsymbol{n}$ is the unit vector along the edge.

The dynamics of the solid are coupled to that of the fluid by transferring force density from solid nodes to the adjacent fluid nodes and advecting the solid with the local velocity of the fluid by using the "spreading" and the "interpolation" of the standard IBM (Boffi et al., 2007). Validation of the numerical scheme and further technical details of our implementation are given in (Cheikh et al., 2023). All simulation code is publicly available at the Doubrovinski lab GitHub link https://github.com/doubrovinskilab/anillin_code. Note that this code depends on additional files available at https://github.com/doubrovinskilab/cantilever_embryo_rheology/tree/main/PETSc_FEM_Code/FEM_Code.

Development • Supplementary information

The geometry and the parameters of the 3D model

The geometry of the model is illustrated in Figure 4. To reduce computational time, we take advantage of reflection symmetry of the embryo and only simulate left half of gastrula (by imposing reflecting boundary condition along the dorso-ventral axis as well, see Figure 4). We also take advantage of the fact that the cross-sectional shape of the embryo during ventral furrow formation is constant for most of the length of the embryo, varying only at the round poles. In our previous work, we studied the pattern of tissue deformation during ventral furrow formation using particle imaging velocimetry (He et al., 2014), and showed that the tissue deformation is actually 2D in nature (there is no flow component along the AP axis) for the vast majority of the ventral furrow. Therefore, we simulate what is essentially a cylindrical embryo by simulating a one-cell-thick cross-section with periodic boundary conditions on both cross-sectional faces. The outer solid boundary represents the rigid vitelline membrane where no-slip boundary condition is imposed. The cellular layer is separated from the model vitelline membrane by a thin layer of perivitelline fluid whose viscosity is set to that of water. Cells are hexagonal prisms whose faces are elastic sheets modelled as a set of inter-connected springs as described in the previous section. Cytoplasm and yolk are both modelled as Newtonian fluid with a viscosity of 1000 cP, as previously measured (Doubrovinski et al., 2017; Selvaggi et al., 2018). The dimensions of cells and their geometries are taken directly from experimental measurements.

Elasticities of cellular edges are taken from measurement data in our recent work where cellular edges of a developing embryo were directly probed mechanically, using bendable cantilevers ((Cheikh et al., 2023); the dimensional constants were taken specifically from Simulation 4 in Figure 7c'' therein). In fact, the model used in the present paper is the same as that described in (Cheikh et al., 2023), except for two additional features. (1) Active forces are imposed on cellular edges to drive tissue folding and (2) basal elasticity in this model is set equal to the lateral rather than basal elasticity from our model in (Cheikh et al., 2023). The reason for this choice is that the previous model described a cellularizing embryo where the basal surface consists of a hexagonal grid of cell edges that are highly enriched with actin. During cellularization, these hexagons shrink until they close off the basal sides of the cells, at which point the enriched actin disappears. Therefore we expect the basal surfaces during ventral furrow formation to be molecularly and mechanically equivalent to the lateral rather than basal surfaces during cellularization. Finally, active stresses (tensions) in the different cellular domains (apical, lateral, and basal) are listed and explained in the caption of the Supplemental Figure S8.

Simplified (2D) model

The computational approach used for the simplified two-dimensional simulations was completely analogous to the one used in 3D simulations, but the geometrical and parameters were chosen differently for simplicity. The computational domain was chosen as a disc representing a cross section through the embryo, with no-slip condition imposed at the boundary (the model vitelline membrane. Membrane elasticity was assumed to be the same in all cellular domains and uniform throughout the embryo. The interior of the embryo (cytoplasm and yolk compartments) was described as a Newtonian fluid of fixed viscosity. Initially, the surface of the embryo is separated from the model vitelline membrane by a gap of uniform thickness (perivitelline space) filled with model perivitelline fluid, described as a relatively inviscid Newtonian fluid. Contractile stresses were imposed on a subset of membranes within the mesoderm (both apical and lateral). Simulations were done using a combination of Finite Element Method and Immersed Boundary Method, completely analogously to the 3D case, except adopting the methods to the two-dimensional geometry in the obvious way. Model parameters used in the different simulations shown in Figure 3 follow; we list these parameters in arbitrary units since they don't correspond to the actual measured values. Simulation of the "wild type" embryo in Figure 3a: radius of the embryo was 117, thickness of the peri-vitelline space is 3. The embryo comprised 101 initially identical cells of height 35. The viscosity of the cytoplasm was 1, viscosity of the perivitelline space was 0.01. The elasticity of springs discretizing cellular boundaries was 0.008. "Mesoderm" was assumed to comprise 17 cells

Development • Supplementary information

whose "apical" and "lateral" membranes were subjected to active stresses (constant force dipoles as was described for the 3D case). The ratio of apical to lateral active stresses in the mesoderm was fixed to 12. Active stresses were ramped linearly according to $3.3 \cdot 10^{-8}\, t + 2 \cdot 10^{-5}$ (in the apical sides of mesodermal cells; stresses in the lateral membranes of the mesoderm had a 12-fold smaller value). All parameters in simulations from Figure 3B-E are the same as in Figure 3A except for the changes listed in the corresponding figure caption. In the simplified 2D model, all parameter values are given in arbitrary units since the model is only meant to serve a proof of principle, while the 3D model incorporates the experimentally measured dimensional quantities. All simulation code is available under https://github.com/doubrovinskilab/anillin_code.

Development • Supplementary information

Supplemental References

Boffi, D., Gastaldi, L., Heltai, L., 2007. Numerical stability of the finite element immersed boundary method. Math Mod Meth Appl S 17, 1479-1505.
Cheikh, M.I., Tchoufag, J., Osterfield, M., Dean, K., Bhaduri, S., Zhang, C., Mandadapu, K.K., Doubrovinski, K., 2023. A comprehensive model of Drosophila epithelium reveals the role of embryo geometry and cell topology in mechanical responses. Elife 12.
Doubrovinski, K., Swan, M., Polyakov, O., Wieschaus, E.F., 2017. Measurement of cortical elasticity in Drosophila melanogaster embryos using ferrofluids. Proc Natl Acad Sci U S A 114, 1051-1056.
He, B., Doubrovinski, K., Polyakov, O., Wieschaus, E., 2014. Apical constriction drives tissue-scale hydrodynamic flow to mediate cell elongation. Nature 508, 392-396.
Peskin, C.S., 1972. Flow Patterns around Heart Valves - Numerical Method. J Comput Phys 10, 252-&.
Peskin, C.S., Printz, B.F., 1993. Improved Volume Conservation in the Computation of Flows with Immersed Elastic Boundaries. J Comput Phys 105, 33-46.
Rauzi, M., Verant, P., Lecuit, T., Lenne, P.F., 2008. Nature and anisotropy of cortical forces orienting Drosophila tissue morphogenesis. Nat Cell Biol 10, 1401-1410.
Selvaggi, L., Pasakarnis, L., Brunner, D., Aegerter, C.M., 2018. Magnetic tweezers optimized to exert high forces over extended distances from the magnet in multicellular systems. Rev Sci Instrum 89, 045106
Seung, H.S., Nelson, D.R., 1988. Defects in Flexible Membranes with Crystalline Order. Phys Rev A 38, 1005-1018.

Development • Supplementary information
