# Supplementary material for: Viscous shear is a key force in Drosophila ventral furrow morphogenesis
Source: Development. 2024 Nov 15;151(22):dev202892. doi: 10.1242/dev.202892 (PMC11586522; doi:10.1242/dev.202892)
Supplement: Supplementary file 1 [file develop-151-202892_review_history.pdf]

Viscous shear is a key force in Drosophila ventral furrow morphogenesis

Amanda Nicole Goldner, Mohamad Ibrahim Cheikh, Miriam Osterfield and Konstantin Doubrovinski
DOI: 10.1242/dev.202892

Editor: Thomas Lecuit

Review timeline
Original submission: 20 March 2024
Editorial decision: 15 May 2024
First revision received: 22 August 2024
Accepted: 9 October 2024

Original submission

First decision letter

MS ID#: DEVELOP/2024/202892

MS TITLE: Viscous shear is a key force in Drosophila ventral furrow morphogenesis

AUTHORS: Amanda Nicole Goldner, Mohamad Ibrahim Cheikh, Miriam Osterfield, and Konstantin Doubrovinski

I apologize for the delay in getting back to you. I have now received all the referees' reports on the above manuscript, and have reached a decision. The referees' comments are appended below, or you can access them online: please go to BenchPress and click on the 'Manuscripts with Decisions' queue in the Author Area.

As you will see, the referees express strong interest in your work, but have some criticisms and recommend a revision of your manuscript before we can consider publication. If you are able to revise the manuscript along the lines suggested, which may involve further experiments, I will be happy receive a revised version of the manuscript. Your revised paper will be re-reviewed by one or more of the original referees, and acceptance of your manuscript will depend on your addressing satisfactorily the reviewers' major concerns. Please also note that Development will normally permit only one round of major revision. If it would be helpful, you are welcome to contact us to discuss your revision in greater detail. Please send us a point-by-point response indicating your plans for addressing the referees' comments, and we will look over this and provide further guidance.

Please attend to all of the reviewers' comments and ensure that you clearly highlight all changes made in the revised manuscript. Please avoid using 'Tracked changes' in Word files as these are lost in PDF conversion. I should be grateful if you would also provide a point-by-point response detailing how you have dealt with the points raised by the reviewers in the 'Response to Reviewers' box. If you do not agree with any of their criticisms or suggestions please explain clearly why this is so.

Reviewer 1

Advance summary and potential significance to field

© 2024. Published by The Company of Biologists under the terms of the Creative Commons Attribution License (https://creativecommons.org/licenses/by/4.0/).

In their manuscript, Goldner et al discuss the process of ventral furrow formation (VFF) during Drosophila morphogenesis. While it was previously assumed that cell volume conservation was needed for VFF to successfully proceed, the authors show experimentally and through modeling that this is not the case.

Overall, I find the manuscript well written and convincing. I feel it is an important addition to the field and recommend its publication.

Comments for the author

comments:

1. From the experiment the authors showed in Figure S7/Movie S1, I found it not entirely clear yet whether cell cytoplasm is viscous or behaves just as yield stress solid.
a) Specifically, do the authors know how the force they applied in the experiment with the magnetic bead (Figure S7/Movie S1) compares to the order of magnitude of the forces driving VFF? More precisely, what needs to be compared are local stress tensors. In the bead experiment, there is likely an inhomogeneous distribution of the stress, which will be largest at the magnetic bead surface. Meanwhile, during unperturbed VFF the stresses are likely somewhat more homogeneous. Unless I overlooked something, this question also did not seem to have been addressed in any of the articles cited in this context (Doubrovinski, 2017; He, 2014; Selvaggi, 2018).
b) If the force applied to the magnetic bead was substantially larger than the forces driving VFF, it would be interesting to experimentally probe also by applying smaller forces, and see if some elastic behavior is found. (I do not want to make this a strict requirement, as the manuscript is already quite convincing as it is.)

2. More out of interest: Are the lateral membranes even required for invagination? One would guess that without lateral membrane the invagination would be less pronounced but it would appear. A check using the model would be quick, but I do not know how feasible an experiment would be. (I think neither is really required for the publication.)

minor comments:

3. The formulation "inviscid Newtonian fluid of fixed (low) viscosity" is a little confusing. If I understood correctly, there is a finite, but low viscosity, so the authors are not considering the inviscid limit. I would propose to just drop the word inviscid (as it is redundant anyways).

4. In the 2D model, there seem to be only two time scales: the time scale of viscous deformation (e.g. quotient of driving forces and viscosity) and the time scale of ramping up of the active forces. It seems very much expected that slowing down viscous deformation and increasing ramping up speed yields the very same result, right? I think it would be good to make this even clearer in the explanation, so Fig. 3E is really only a sanity check.

5. "Allowing cell rearrangements would effectively decrease the elasticity of the apical surface, which could allow for deeper furrow invagination." - I have doubts about this statement, and at the very least it should be explained better. Cell rearrangements can help dissipate anisotropic stresses, but apical constriction in this system is quite possibly driven by isotropic active stresses.

6. 3D model: To be honest, given that the 3D model anyways assumes homogeneity along the AP axis, it is not clear to me what it actually teaches us beyond the 2D model. I know more realistic parameters have been applied, but one could also do this in the 2D model, right?

7. In the last paragraph before the discussion section, I find the discussion about the process being at mechanical equilibrium a tad too vague. A system is at mechanical equilibrium when it is at force balance (including both elastic *and* frictional forces). As the authors correctly state, the system is far in the overdamped limit, which implies mechanical equilibrium always. Yet, it seems the authors want to say something more here, e.g. that the viscous stresses relax away quickly to a mechanical equilibrium *between elastic forces only*.

© 2024. Published by The Company of Biologists under the terms of the Creative Commons Attribution License (https://creativecommons.org/licenses/by/4.0/).

typos:

- "Anillin mutants additionally present with membrane degradation and nuclear displacement"

Reviewer 2

Advance summary and potential significance to field

Goldner et al. examine the mechanisms that underlie ventral furrow formation in Drosophila with a particular interest in testing whether viscous shear contributes to the force mechanisms that drive mesoderm invagination. They employ a genetic background (Anillin/scraps shRNA) that fails to seal off the basal membrane, leaving cells open at their basal ends (lateral cell membranes also later degenerate in this background), and two different computational models. There are interesting ideas being explored in this work, but conclusions also largely rest on the interpretation and fidelity of the models.

A few major and minor comments are below:

Comments for the author

1) The anillin data is interesting, but could be made more clear. What is the x-axis in the length measurements? It seems like it should be a time component, but it wasn't obvious what these measurements are. Also, the legend says differences are statistically significant, but how these were tested (and what exactly was tested) is unclear. Having a box and whisker plots of average lengths of wild-type and scraps at key time points with significances would be useful. Also, are the control images really "wild-type". Similar driver levels, etc should serve as the control (optimally, driving a shRNA construct that does not affect VF formation would be the best control).

2) It seemed that the magnetic bead experiment could be problematic – it appeared that the bead was held in an apical position for 20-30 seconds, so how could elastic recoil be measured? Previous work has suggested that elastic responses and relaxation in the early embryo occur very rapidly, on the time scale of a few seconds, so is this an appropriate conclusion? This is a key point for the authors analysis.

3) On a more minor note, the characterization of the Fierling and Rauzi paper should be more carefully stated. I don't believe they specifically attributed the phenotypes to a lack of compartmentalization, but instead said a variety of potential causes (lack of a basal membrane/basal force generation compartmentalization, etc) could be driving the observed behaviors.

4) Top of page 8 – "these findings suggest that Anillin plays a role in VF formation". This does not seem accurate, anillin appears to affect cell integrity rather than playing a direct role in the VF – or is this effect confined to the VF?

5) Some aspects of the modeling were not well-described – for example, what was the justification for applying equal stress to both lateral and apical membranes? (myosin networks are apical in location).

6) Similar to above, why were certain values chosen? The ramping speed was decreased by 33 fold at one point in their modeling, but no justification for why this value was chosen is given. Were other values explored? Was this the value that gave the most robust response, or best matched experimental expectations? This is a consistent concern throughout the described modeling approaches.

7) Given that the conclusions rest on computational models, it seems difficult to make some of the strong statements about how VF occurs in vivo, such as "is driven by shear forces" (Discussion, pg 15). Perhaps these could be more carefully worded.

© 2024. Published by The Company of Biologists under the terms of the Creative Commons Attribution License (https://creativecommons.org/licenses/by/4.0/).

<u>Reviewer 3</u>

Advance summary and potential significance to field

The manuscript investigates the role of cell volume conservation during ventral furrow formation. This major early folding event in Drosophila is a model for other tissue morphogenesis processes, and therefore of broad interest to developmental biologists and physicists. Using actin binding anillin knockdown via scra RNAi, the authors describe delays in cellularization, and document reduced level of basal membrane formation. This phenotype is accompanied by subtle differences in the pattern of junction lengths compared to the wild type.
Cross sections clearly show signs of ventral furrow formation in the mutant. The tissue remains folded despite lateral membrane degradation, suggesting folding is not a transient observation. Earlier investigations by the lab have shown elegantly that the cytoplasm prior to cellularization is viscous. This lets the authors speculate the cytoplasm inside cells is also viscous, and a physical test of this reasonable hypothesis is proposed. In lieu of further experimental tests, a simplified theoretical model is presented, to investigate putative mechanisms driving furrow formation. Here, viscosity as an effective volume constraint is somewhat ad hoc introduced. Folds in this two-dimensional model and a three-dimensional model extension still form given sufficiently high viscosity. The authors conclude that viscous shear forces and basal membrane formation play complementary mechanical roles to support ventral furrow formation.

The Manuscript has potential to combine experiment and theory. But improved connection between theory and experiment, and better documentation of experiments are required to be convincing.

Comments for the author

Below is a summary of major points that reduce my enthusiasm for the manuscript somewhat. In particular item 3 is concerning.

1) Viscosity test: A magnetic bead half the size of the cells is forced through the cell with a magnet. The manuscript describes lack of recoil, which may be consistent with viscosity. While viscosity is a reasonable assumption, this specific experiment is somewhat challenging to interpret given the information provided.
Moving a bead half the size of a cell through the entire cell body is likely to disturb the cell in many ways. It is unclear how the cytoskeleton (microtubules, actin, myosin) interplays with the bead. It may be possible the bead gets trapped by the cytoskeleton, and therefore does not recoil. It is also possible the bead destroys these structures. What forces are applied to the bead as it gets dragged through the cell? Could cytoplasmic flow be visualized using microbeads?

2) It remains unclear how the annillin phenotype is established. The known function of annillin should be better described. actin dynamics is affected during cellularization: are the dynamics of the cytoskeleton unchanged in these mutants later on? Especially the pulsatile activity on the apical surface but also microtubules are of interest. A better characterization of actin dynamics would be welcomed.

3) A model is a useful bridge between experiments and hypotheses, especially when it provides testable predictions. It is not clear the viscosity based model presented here is consistent with experimental data shown in the first two figures. Based on intuition, the authors propose viscosity leads to an effective volume constraint. Figures 3 and 4 show plenty of cross sections from simulations, but a quantitative connection of reported observables even for cell lengths shown in figures 1 and 2 remains missing. We are further puzzled by other cell shape indicators from data. Figure 1I and 1C show late furrows in cross section. The control furrow in 1C shows membrane is curved in a monotonous way. As expected for wild type, this suggests pressure inside cells is systematically graded across the tissue as it folds. Pressure in cells is consistent with a volume constraint. The picture is very different in the mutant. Scra RNAi shows essentially straight membranes. Some few membranes appear subtly curved in both directions, suggesting pressure plays a minor role here (Figure 2B’). The membrane shape of scra RNAi mutants further appears inconsistent with the cell shapes presented for the no basal membrane scenarios discussed in

© 2024. Published by The Company of Biologists under the terms of the Creative Commons Attribution License (https://creativecommons.org/licenses/by/4.0/).

figures 3 and 4. The simulations predict a similarly curved interface as in the case with basal membranes present. Further quantitative tests of the model, comparing membrane curvatures, and visualizing the velocity gradient inside a cell would be a very welcome quantitative confirmation of this model.

4) The argument about realistic models remains difficult to follow. Calling the model realistic is particularly prone to misinterpretation and should be changed. The description of the model is too brief to justify how the three dimensional system actually is realistic. In particular, the physical phenomena do not appear any different from prior cases. Much of the physics of cells, such as microtubules also appear neglected. It may be better to rename this model, to reduce ambiguity.

First revision

Author response to reviewers' comments

Reviewer 1 Advance summary and potential significance to field

In their manuscript, Goldner et al discuss the process of ventral furrow formation (VFF) during Drosophila morphogenesis. While it was previously assumed that cell volume conservation was needed for VFF to successfully proceed, the authors show experimentally and through modeling that this is not the case.

Overall, I find the manuscript well written and convincing. I feel it is an important addition to the field and recommend its publication.

Reviewer 1 Comments for the author

comments:

1. From the experiment the authors showed in Figure S7/Movie S1, I found it not entirely clear yet whether cell cytoplasm is viscous or behaves just as yield stress solid.
a) Specifically, do the authors know how the force they applied in the experiment with the magnetic bead (Figure S7/Movie S1) compares to the order of magnitude of the forces driving VFF? More precisely, what needs to be compared are local stress tensors. In the bead experiment, there is likely an inhomogeneous distribution of the stress, which will be largest at the magnetic bead surface. Meanwhile, during unperturbed VFF the stresses are likely somewhat more homogeneous. Unless I overlooked something, this question also did not seem to have been addressed in any of the articles cited in this context (Doubrovinski, 2017; He, 2014; Selvaggi, 2018).
b) If the force applied to the magnetic bead was substantially larger than the forces driving VFF, it would be interesting to experimentally probe also by applying smaller forces, and see if some elastic behavior is found. (I do not want to make this a strict requirement, as the manuscript is already quite convincing as it is.)

We fully agree with Reviewer 1: the possible effects of yield stress complicate the interpretation of experiments from Figure S7. Although the approach suggested in (a) is reasonable, the approach suggested in (b) seems even stronger, so we have addressed this concern by probing only with endogenous forces. Specifically, we have now performed additional experiments where fluorescent polystyrene microspheres with a diameter of 0.5 microns were injected into the yolk during mid- to late cellularization stage. Next, those beads were imaged using spinning disk confocal microscopy for 10 min. Using Imaris software, we tracked beads over time and analyzed their trajectories. By plotting mean square displacement (MSD) of those beads as a function of time, we found that those beads underwent unrestricted diffusion during the entire time-period of 10 min. Specifically, as is now shown in the new figure S8, the MSD curve is well approximated by a straight line. These data are consistent with the cytoplasm being entirely viscous, and not elastic. Were those beads confined by permanent elastic structures, their diffusion due to random fluctuation would be restricted, causing the MSD curve to flatten out over time approaching a constant. These data suggest that either (1) fluctuations inherently present in the cytoplasm are strong enough to

© 2024. Published by The Company of Biologists under the terms of the Creative Commons Attribution License (https://creativecommons.org/licenses/by/4.0/).

overcome the yield stress, or (2) that the cytoplasm in viscous and there is no yield stress to overcome. We have also included a discussion of this additional evidence at the top of page 10.

2. More out of interest: Are the lateral membranes even required for invagination? One would guess that without lateral membrane the invagination would be less pronounced but it would appear. A check using the model would be quick, but I do not know how feasible an experiment would be. (I think neither is really required for the publication.)

This is indeed an interesting question. In the parameter sweeps we did with our 2D model, we found that lateral membranes (or more accurately, sufficiently high lateral membrane tensions) were in fact required for invagination. We did not include this result in this manuscript partly since it seemed somewhat separate from the main topic at hand. More to the point, though, this question has been answered directly in the experimental literature. In the slam dunk double mutant, cellularization fails and lateral membranes never form. Although slam dunk double mutants do undergo apical constriction, this does not result in any invagination of the tissue. (Please see He, Martin, and Wieschaus, Development (2016) 143:2417 if you are interested in further details.)

minor comments:

3. The formulation "inviscid Newtonian fluid of fixed (low) viscosity" is a little confusing. If I understood correctly, there is a finite, but low viscosity, so the authors are not considering the inviscid limit. I would propose to just drop the word inviscid (as it is redundant anyways).

Thank you for pointing this out - the word “inviscid” has now been removed.

4. In the 2D model, there seem to be only two time scales: the time scale of viscous deformation (e.g. quotient of driving forces and viscosity) and the time scale of ramping up of the active forces. It seems very much expected that slowing down viscous deformation and increasing ramping up speed yields the very same result, right? I think it would be good to make this even clearer in the explanation, so Fig. 3E is really only a sanity check.

Reviewer 1 is correct; indeed, simulation in Fig. 3E is just a sanity check. We have modified the wording in the main text (page 12) to try to get this idea across, and we have also added a more detailed explanation to the caption of figure 3E.
We realize that a way to demonstrate this more formally would have been to re-scale model parameters to make them non-dimensional. This would show that changing the time-scale of force ramping is indeed equivalent to changing viscosity. However, since our 3D modeling is based on measured parameters, we preferred not to re-scale parameter values and work with dimensional numbers instead.

5. "Allowing cell rearrangements would effectively decrease the elasticity of the apical surface, which could allow for deeper furrow invagination." - I have doubts about this statement, and at the very least it should be explained better. Cell rearrangements can help dissipate anisotropic stresses, but apical constriction in this system is quite possibly driven by isotropic active stresses.

We understand Reviewer 1's point. We have now weakened this statement to “Allowing for cell rearrangements could possibly result in closer agreement between data and theory.”

6. 3D model: To be honest, given that the 3D model anyways assumes homogeneity along the AP axis, it is not clear to me what it actually teaches us beyond the 2D model. I know more realistic parameters have been applied, but one could also do this in the 2D model, right?

Since our model is meant to reproduce quantitative details of measurement data, we decided that a 3D model is necessary for the following reason: the 2D model is not actually its limit. Strictly, the 3D counterpart of our 2D model would have cells that are infinitely long along the dimension extending into the plane of the page. While this may be a reasonable approximation, it does not quite correspond to the situation we would ideally like to study. In order to make cells have equal dimensions along the antero-posterior and medio-lateral axes, a 3D model is necessary.
To make this point more clear, we have now included a brief note to this effect on page 12, following the discussion of the 2D model.

© 2024. Published by The Company of Biologists under the terms of the Creative Commons Attribution License (https://creativecommons.org/licenses/by/4.0/).

7. In the last paragraph before the discussion section, I find the discussion about the process being at mechanical equilibrium a tad too vague. A system is at mechanical equilibrium when it is at force balance (including both elastic *and* frictional forces). As the authors correctly state, the system is far in the overdamped limit, which implies mechanical equilibrium always. Yet, it seems the authors want to say something more here, e.g. that the viscous stresses relax away quickly to a mechanical equilibrium *between elastic forces only*.

We agree with Reviewer 1 that our arguments on this point may appear somewhat vague. To avoid confusion, we have removed the last two sentences of this paragraph, limiting our claims to the statement that the time scale of ventral furrow formation is determined by the rate at which active forces build up, and not by the rate at which the tissue responds to those forces. We believe this is strongly supported by our measurements and modeling. Specifically, it is certainly the case in our model (since turning the active forces on at once results in invagination taking only ~5 sec), and since model parameters are constrained by measurements (at least at the level of orders of magnitude), we propose that this ought to hold in vivo.

typos:
- "Anillin mutants additionally present with membrane degradation and nuclear displacement"

Thank you for catching this - the typo has been corrected.

***** Reviewer 2 Advance summary and potential significance to field
Goldner et al. examine the mechanisms that underlie ventral furrow formation in Drosophila with a particular interest in testing whether viscous shear contributes to the force mechanisms that drive mesoderm invagination. They employ a genetic background (Anillin/scraps shRNA) that fails to seal off the basal membrane, leaving cells open at their basal ends (lateral cell membranes also later degenerate in this background), and two different computational models. There are interesting ideas being explored in this work, but conclusions also largely rest on the interpretation and fidelity of the models.

A few major and minor comments are below:

Reviewer 2 Comments for the author
1) The anillin data is interesting, but could be made more clear. What is the x-axis in the length measurements? It seems like it should be a time component, but it wasn't obvious what these measurements are. Also, the legend says differences are statistically significant, but how these were tested (and what exactly was tested) is unclear. Having a box and whisker plots of average lengths of wild-type and scraps at key time points with significances would be useful. Also, are the control images really "wild-type". Similar driver levels, etc should serve as the control (optimally, driving a shRNA construct that does not affect VF formation would be the best control).

The x-axis in those plots refers to the position of the cell: the number counts how many cells away a given membrane is from the ventral midline. The midline is identified as the center of the pattern of Snail expression. We thank Reviewer 2 for pointing out this lack of clarity and we have now included a clarifying sentence in the corresponding figure caption.
The details of our statistical analysis are given in the "Statistical analysis of immunostaining results" subsection of the Methods section. We have now included a reference to this section in the caption of Figure 1.
In response to this comment, we have also substituted box-and-whiskers plots for our previous plots of mean +/- standard deviation.
Reviewer 2 is correct - The controls shown throughout the manuscript are not actually wild type, but rather are the same genotype as in the experiment but without the scra RNA (TRiP.GL01269) component. We have now labeled this more clearly in the figures and text (either as "no RNAi" or as "control"), and described this in the Methods. Additionally, we have now repeated some of the analysis with a shRNA construct for a gene that is not expected to affect VF formation or any early morphogenesis events (Loxl1). These experiments closely resemble the results seen in our no RNAi controls, and are shown in the new Supplementary figure panels S2C-D.

© 2024. Published by The Company of Biologists under the terms of the Creative Commons Attribution License (https://creativecommons.org/licenses/by/4.0/).

2) It seemed that the magnetic bead experiment could be problematic – it appeared that the bead was held in an apical position for 20-30 seconds, so how could elastic recoil be measured? Previous work has suggested that elastic responses and relaxation in the early embryo occur very rapidly, on the time scale of a few seconds, so is this an appropriate conclusion? This is a key point for the authors analysis.

Yes, it is certainly true that cellular cytoplasm can exhibit elastic responses on short time-scales of seconds (as was previously demonstrated in e.g. Lau et al. PRL 2003 and Hoffman et al. PNAS 2006 for tissue culture cells). However, developmental processes we are interested in take place on a time scale of minutes, which is two orders of magnitude slower. Elastic response that dissipates on a time scale of seconds is irrelevant to processes that require minutes.
However, in response to this and other Reviewer concerns about this experiment, we have now included a complementary experiment characterizing the diffusion of (non-magnetic) beads; this new data also strongly indicates that the cytoplasm is viscous at this stage. See new Supplementary Figure S8 and our reply to Reviewer 1's first point for more information.

3) On a more minor note, the characterization of the Fierling and Rauzi paper should be more carefully stated. I don't believe they specifically attributed the phenotypes to a lack of compartmentalization, but instead said a variety of potential causes (lack of a basal membrane/basal force generation, compartmentalization, etc) could be driving the observed behaviors.

We thank Reviewer 2 for this comment. Now, we have added the following parenthetical statement to the relevant discussion: "although additional factors associated with the genetic background of the acellular embryo may be contributing to the phenotype as well".

4) Top of page 8 – "these findings suggest that Anillin plays a role in VF formation". This does not seem accurate, anillin appears to affect cell integrity rather than playing a direct role in the VF – or is this effect confined to the VF?

We thank Reviewer 2 for pointing this out. The phrasing has been changed to "Anillin affects cell behavior during ventral furrow formation".

5) Some aspects of the modeling were not well-described – for example, what was the justification for applying equal stress to both lateral and apical membranes? (myosin networks are apical in location).

Apical and lateral stresses are not equal in either the 2D or the 3D model. Specifically, the ratio of apical to lateral stresses was set to 12 and 10 in the 2D and the 3D models respectively. This is stated in the description of the 2D model (very end of the Appendix) and in the caption of the old figure S8 (which is now S9). We are not entirely certain about which modeling details are not included in the manuscript. All model parameters are listed (in either the main text or the appendix), and all code has been made publicly available. If Reviewer 2 could make more specific suggestions about which specific additional details need to be described, we will be happy to do so.

6) Similar to above, why were certain values chosen? The ramping speed was decreased by 33 fold at one point in their modeling, but no justification for why this value was chosen is given. Were other values explored? Was this the value that gave the most robust response, or best matched experimental expectations? This is a consistent concern throughout the described modeling approaches.

Here Reviewer 2 is referring to simulation results in Figures 3B-E. The purpose of those simulations was to demonstrate a key conclusion: the smaller the rate of invagination, the less its extent. The factor of 33 is completely arbitrary: making ramping speed smaller will result in an even smaller extent of invagination. Conversely, making ramping speed higher will increase the extent of invagination, ultimately making is as large as in Figure 3B. We emphasize that the 2D simulation is just a toy model to illustrate this very point: the smaller the effects of viscosity, the smaller the extent of invagination. Effects of viscosity can be varied in two major ways (which are essentially equivalent, as was rightfully noted by Reviewer 1): either making viscosity smaller, or decreasing the ramping speed decreases the overall contribution of the viscous forces. To try to more clearly

© 2024. Published by The Company of Biologists under the terms of the Creative Commons Attribution License (https://creativecommons.org/licenses/by/4.0/).

indicate that the 33-fold ramp was randomly chosen as some larger number, we have changed the phrasing to "greatly decreased the ramping speed (by 33-fold)".

In the 3D model, three types of parameters were used. (1) The initial geometry of cells. This was taken directly from measurements. (2) Material properties of the different cellular structures and the cytoplasm. All values were as determined in our previous measurements (Cheikh et al 2023 and Doubrovinski et al 2018). (3) Finally, the distribution of contractile forces was chosen in accordance with the previously reported myosin distribution to approximate the observed time-course of tissue invagination. We did not perform systematic parameter sweeps in the 3D model: our simulations are fairly time-expensive requiring approximately three weeks of simulation time on 100 processor cores. It is certainly true that the exact distribution and the time-course of myosin-driven tensions are not known experimentally. However, crucially, our key conclusion does not really rely on this information. Our claim is that if folding happens on the experimentally relevant time scale, the experimentally measured viscosity value is sufficient to maintain volume conservation even when cells are open on their basal side. There is no obvious reason why this conclusion would be sensitive to the exact spatial distribution of contractile forces (although it will certainly be sensitive to the rate of increase of those forces). We emphasize that our major claim is only about the relative orders of magnitude of the relevant effects: if folding is as fast as observed and if viscosity is as previously measured, the loss of cytoplasm through basal holes will be small enough to ensure robust invagination. We have now added the following comment to the discussion of the 3D model on page 13:
“Note that the exact distribution of contractile forces is not known experimentally. However, we do not expect our key conclusions to be sensitive to the exact spatial distribution of those force, but only to the speed at which those forces are ramped.”

7) Given that the conclusions rest on computational models, it seems difficult to make some of the strong statements about how VF occurs in vivo, such as “is driven by shear forces” (Discussion, pg 15). Perhaps these could be more carefully worded.

Thank you for pointing this out: this last phrase was removed to avoid overstatements.

***** Reviewer 3 Advance summary and potential significance to field
The manuscript investigates the role of cell volume conservation during ventral furrow formation. This major early folding event in Drosophila is a model for other tissue morphogenesis processes, and therefore of broad interest to developmental biologists and physicists. Using actin binding anillin knockdown via scra RNAi, the authors describe delays in cellularization, and document reduced level of basal membrane formation. This phenotype is accompanied by subtle differences in the pattern of junction lengths compared to the wild type.
Cross sections clearly show signs of ventral furrow formation in the mutant. The tissue remains folded despite lateral membrane degradation, suggesting folding is not a transient observation. Earlier investigations by the lab have shown elegantly that the cytoplasm prior to cellularization is viscous. This lets the authors speculate the cytoplasm inside cells is also viscous, and a physical test of this reasonable hypothesis is proposed. In lieu of further experimental tests, a simplified theoretical model is presented, to investigate putative mechanisms driving furrow formation. Here, viscosity as an effective volume constraint is somewhat ad hoc introduced. Folds in this two-dimensional model and a three-dimensional model extension still form, given sufficiently high viscosity. The authors conclude that viscous shear forces and basal membrane formation play complementary mechanical roles to support ventral furrow formation.

The Manuscript has potential to combine experiment and theory. But improved connection between theory and experiment, and better documentation of experiments are required to be convincing.

Reviewer 3 Comments for the author
Below is a summary of major points that reduce my enthusiasm for the manuscript somewhat. In particular item 3 is concerning.

1) Viscosity test: A magnetic bead half the size of the cells is forced through the cell with a magnet. The manuscript describes lack of recoil, which may be consistent with viscosity. While viscosity is a reasonableassumption, this specific experiment is somewhat challenging to interpret given the information provided.

© 2024. Published by The Company of Biologists under the terms of the Creative Commons Attribution License (https://creativecommons.org/licenses/by/4.0/).

Moving a bead half the size of a cell through the entire cell body is likely to disturb the cell in many ways. It is unclear how the cytoskeleton (microtubules, actin, myosin) interplays with the bead. It may be possible the bead gets trapped by the cytoskeleton, and therefore does not recoil. It is also possible the bead destroys these structures. What forces are applied to the bead as it gets dragged through the cell? Could cytoplasmic flow be visualized using microbeads?

We fully agree with Reviewer 3's concerns, which mirror those raised by Reviewer 1 in their 1st point. Please see our response to them above for a detailed reply. In summary, though, following the suggestion by Reviewer 3 here, we have now performed additional experiments where fluorescent polystyrene microspheres (beads) were injected during mid- to late cellularization stage. We then tracked and analyzed beads which had entered the cellular interiors by the beginning of the time-lapse imaging . Analysis of mean square displacement (MSD) as a function of time showed that the beads underwent unrestricted diffusion during the entire time-period of 10 minutes, and the MSD curve is well approximated by a straight line. These data are consistent with the cytoplasm being entirely viscous, and not elastic. These new data are shown and described in a new Supplementary Figure S8, and we have included a discussion of this additional evidence at the top of page 10.

2) It remains unclear how the annillin phenotype is established. The known function of annillin should be better described. actin dynamics is affected during cellularization: are the dynamics of the cytoskeleton unchanged in these mutants later on? Especially the pulsatile activity on the apical surface but also microtubules are of interest. A better characterization of actin dynamics would be welcomed.

From previous publications, it is known that anillin is specifically localized to the leading (basal) edge of the growing membranes during all phases of cellularization, and that loss of anillin results in loss of actin, myosin, and septin localization to these specific structures. We have now added a sentence to this effect to the first paragraph of the Results section, along with the relevant references.
We are less clear on the reason behind the second part of this comment. We didn't characterize the pulsatile activity on the apical surface for two reasons: 1) anillin isn't localized there, so there is no reason to expect an effect there (although of course we had not previously pointed this out in our text), and 2) apical constriction occurs normally, so there was no reason to suspect pulsatile activity or any other associated processes were defective. (We do realize that there has been a minor amount of speculation that the pulsatile activity seen during apical constriction may be an epiphenomenon, and if any mutants were found to exhibit normal apical constriction without pulsatile activity, such an observation would support that point of view. However, we feel that looking for such an effect is really outside the scope of this manuscript.)

3) A model is a useful bridge between experiments and hypotheses, especially when it provides testable predictions. It is not clear the viscosity based model presented here is consistent with experimental data shown in the first two figures. Based on intuition, the authors propose viscosity leads to an effective volume constraint. Figures 3 and 4 show plenty of cross sections from simulations, but a quantitative connection of reported observables even for cell lengths shown in figures 1 and 2 remains missing. We are further puzzled by other cell shape indicators from data. Figure 1I and 1C show late furrows in cross section. The control furrow in 1C shows membrane is curved in a monotonous way. As expected for wild type, this suggests pressure inside cells is systematically graded across the tissue as it folds. Pressure in cells is consistent with a volume constraint. The picture is very different in the mutant. Scra RNAi shows essentially straight membranes. Some few membranes appear subtly curved in both directions, suggesting pressure plays a minor role here (Figure 2B'). The membrane shape of scra RNAi mutants further appears inconsistent with the cell shapes presented for the no basal membrane scenarios discussed in figures 3 and 4. The simulations predict a similarly curved interface as in the case with basal membranes present. Further quantitative tests of the model, comparing membrane curvatures, and visualizing the velocity gradient inside a cell would be a very welcome quantitative confirmation of this model.

We thank Reviewer 3 for these suggestions. Previously, we did include a comparison between model and experiment for one key measurement: the relative length of central versus peripheral membranes. In both experiment and model, the peripheral membranes are longer in the control,

© 2024. Published by The Company of Biologists under the terms of the Creative Commons Attribution License (https://creativecommons.org/licenses/by/4.0/).

while the central membranes are longer in the scra RNAi case (Supplemental figure S4). In response to this suggestion though, we have now included a new Supplemental Figure S10 which shows the membrane lengths and membrane curvatures as a function of position for both experiments and the 3D model. As can be easily seen, the model qualitatively captures several features seen in the experiments, although the models are obviously not identical to the experimental results. Interestingly, we find that membrane curvature as a function of position is remarkably similar between control and scra RNAi in both experiment and model. We must admit that we were a little surprised to see this in our measurements, since Reviewer 3's observation that the mutant membranes seemed less curved also appeared that way to us initially. However, on further reflection, we realized that this is probably an optical effect due to the shorter membrane lengths - a long enough curve will turn into a circle, while a shorter curve of the same curvature will not take such a drastic change in trajectory.

4) The argument about realistic models remains difficult to follow. Calling the model realistic is particularly prone to misinterpretation and should be changed. The description of the model is too brief to justify how the three dimensional system actually is realistic. In particular, the physical phenomena do not appear any different from prior cases. Much of the physics of cells, such as microtubules also appear neglected. It may be better to rename this model, to reduce ambiguity.

In response to this concern, we have removed all use of the word "realistic" in describing our 3D model, although we retain that word when discussing ideals for models in general (on page 12 and 14).

The information contained in this message and any attachment is confidential, legally privileged and is intended for the addressee only. Any dissemination, distribution, copying, disclosure or use of this message/attachment or its contents is strictly prohibited and may be unlawful. No contract is intended or implied, unless confirmed by hard copy. If you have received this message in error, please inform the sender and delete it from your mailbox or any other storage mechanism.

The Company Of Biologists Ltd cannot accept liability for any statements made which are clearly the senders' own and not expressly made on behalf of the Company of Biologists Limited or one of their agents.

Second decision letter

MS ID#: DEVELOP/2024/202892

MS TITLE: Viscous shear is a key force in Drosophila ventral furrow morphogenesis

AUTHORS: Amanda Nicole Goldner, Mohamad Ibrahim Cheikh, Miriam Osterfield, and Konstantin Doubrovinski

I apologise for the delay in getting back to you. We received the referees reports, two of them are enthusiastic about the work, while one is a bit more reserved. Considering all comments I am happy to tell you that your manuscript has been accepted for publication in Development, pending our standard publication integrity checks.

Reviewer 1

Advance summary and potential significance to field

In their manuscript, Goldner et al discuss the process of ventral furrow formation (VFF) during Drosophila morphogenesis. While it was previously assumed that cell volume conservation was needed for VFF to successfully proceed, the authors show experimentally and through modeling that this is not the case.

© 2024. Published by The Company of Biologists under the terms of the Creative Commons Attribution License (https://creativecommons.org/licenses/by/4.0/).

Overall, I find the manuscript well written and convincing. I feel it is an important addition to the field and recommend its publication.

Yet, I feel that some intuition about what is actually happening in the model could be better discussed (see my point 3 below).

Comments for the author

1. The authors now write: "One potential such factor might be cell rearrangements, which we do not include in our model. Allowing for cell rearrangements could possibly result in closer agreement between data and theory." They now shortened the second sentence as a results of my former criticism. Yet, it is still not clear how cell rearrangements would help. Do the authors have something specific in mind here? If not, I do not find this statement particularly helpful.

2. I am still not sure I understand the last results paragraph, right before the discussion. The authors write that their "[...] computational experiments provided additional evidence [...] that ventral furrow formation is an adiabatic process." It would be of great help to specify what is meant by "adiabatic" in this context. In physics, this usually means that one time scale is much larger than another time scale. There seem to be essentially two principal time scales in their model: the time scale on which the active stresses are ramped up, and the visco-elastic relaxation time of the system. I suspect that by "adiabatic" the authors want to say that the embryo corresponds to the limit where the visco-elastic relaxation time is much smaller than the time scale of ramping up the force.
However, this seems to be very much inconsistent with figure 3, which shows that when going closer to this limit (Fig. 3D,E), the simulation results suggest smaller VF invagination.

3. Thinking about it, the model without the basal membrane seems to describe the apical membrane as an elastic material, interacting with the viscous fluid. So, after viscous relaxation and at any given active tension value, the total apical length should be given by Ncells*(rest length - final active tension/k). Is this correct? Yet, the total apical length at the end of the simulation seems to be larger than the total apical length in the initial state, suggesting that what we see in this simulation is guided buckling.
a) I did not find a value for the rest length of the apical springs, and neither one for the spring constant. It would be good to list these parameters. Is Ncells*(rest length) indeed larger than the total initial apical length? In this case, what is simulated is indeed guided buckling, it would be good to clearly state that. This would imply that the cells in the scra RNAi embryos are initially not under apical tension, but under compression, which seems somewhat surprising. The lack of effective apical tension in the model seems to be confirmed by the inward curvature of the apical interface in the final state.
b) It would be good to enrich the main text with more of this kind of discussion about what is actually happening in the scra RNAi simulations, to provide more intuition to both physicist and biologist readers.
Both points equally apply to the 3D model.

typos:
- "Samples were imaged for 10 minus using confocal spinning disk microscopy." --> "minutes"

<u>Reviewer 2</u>

Advance summary and potential significance to field

The revision by Goldner et al. addresses several of the reviewer comments – in general, I would say that the (minor) revisions clean up some of the smaller experimental concerns, but do not change the overall impact of the paper. This is a minimalist paper (4 main figures – as a side note, there's more supplementary figures than main, and so why some of the sup figures aren't moved to the main text is unclear), and the level of analysis is not very detailed. However, the finding that anillin-compromised embryos still make ventral furrows is a nice and intriguing finding, while the key conclusions about shear forces and viscous displacements also largely rest on the interpretation and fidelity of the models. Both a 2D and 3D model are presented – I'm not sure why both are

© 2024. Published by The Company of Biologists under the terms of the Creative Commons Attribution License (https://creativecommons.org/licenses/by/4.0/).

needed, and no argument on this is offered in the text. It still seems that, given that elastic forces have been previously measured by several groups in the early embryo, more explanation should be offered than just ignoring the discrepancy between approaches and saying that it's not relevant for their work (without describing why in the manuscript). For me, this remains a borderline manuscript for Development, largely because of the minimal characterization, but it will be interesting to see what the other reviewers conclude.

Comments for the author

The revision by Goldner et al. addresses several of the reviewer comments – in general, I would say that the (minor) revisions clean up some of the smaller experimental concerns, but do not change the overall impact of the paper. This is a minimalist paper (4 main figures – as a side note, there's more supplementary figures than main, and so why some of the sup figures aren't moved to the main text is unclear), and the level of analysis is not very detailed. However, the finding that anillin-compromised embryos still make ventral furrows is a nice and intriguing finding, while the key conclusions about shear forces and viscous displacements also largely rest on the interpretation and fidelity of the models. Both a 2D and 3D model are presented – I'm not sure why both are needed, and no argument on this is offered in the text. It still seems that, given that elastic forces have been previously measured by several groups in the early embryo, more explanation should be offered than just ignoring the discrepancy between approaches and saying that it's not relevant for their work (without describing why in the manuscript). For me, this remains a borderline manuscript for Development, largely because of the minimal characterization, but it will be interesting to see what the other reviewers conclude.

Reviewer 3

Advance summary and potential significance to field

The authors have addressed my concerns, and added valuable explanations as well as additional data that makes the manuscript more convincing than the initial submission.

Comments for the author

The authors have addressed my concerns, and added valuable explanations as well as additional data that makes the manuscript more convincing than the initial submission.

© 2024. Published by The Company of Biologists under the terms of the Creative Commons Attribution License (https://creativecommons.org/licenses/by/4.0/).
